# Plant Identity Shapes Core Microbiomes on Green Roofs

**DOI:** 10.1111/1758-2229.70451

**Published:** 2026-10-30

**Authors:** Katarzyna Larysz, Michał Ludynia, Artur Trzebny, Wiktoria Klencz, Natalia Malcherczyk, Edyta Sierka

**Affiliations:** ^1^ International Environmental Doctoral School in Sosnowiec University of Silesia in Katowice Katowice Poland; ^2^ Faculty of Natural Sciences, Institute of Biology, Biotechnology and Environmental Protection University of Silesia in Katowice Katowice Poland; ^3^ Faculty of Biology, Institute of Molecular Biology and Biotechnology, Molecular Biology Techniques Laboratory Adam Mickiewicz University Poznan Poznan Poland

**Keywords:** amplicon sequencing, coal mine spoil heaps, green roof substrate, microbial biomarkers, microbial diversity patterns, plant functional traits, sterilization‐mediated priority effect

## Abstract

Extensive green roof substrates support plant–microbe interactions that may contribute to ecosystem functioning. Yet the role of plant identity in shaping substrate‐associated microbiomes remains insufficiently resolved beyond *Sedum*‐dominated systems. We examined bacterial and fungal communities associated with three plant species representing contrasting functional groups in sterilized and non‐sterilized green roof substrates, integrating taxonomic differentiation with plant functional traits and predicted metabolic pathways. Plant identity was associated with distinct microbial community patterns in sterilized and non‐sterilized substrates, and these associations varied with the substrate's initial microbial state. LEfSe identified 13 discriminant fungal genera in ITS data and 65 bacterial genera in V4 data; strong discriminant taxa included *Mortierella, Schizothecium*, and *Serendipita* in fungal communities, and *Sphingomonas, Brevundimonas*, and *Tumebacillus* in bacterial communities. *Lotus corniculatus* showed particularly distinct plant‐associated microbial patterns, while differences in predicted metabolic pathways provided a complementary functional perspective on taxonomic differentiation. Integrating plant functional traits with bacterial and fungal community analyses provided an ecological framework for interpreting plant‐associated microbiome differentiation in extensive green roof substrates. Integrating plant functional traits with microbial community analyses provides an ecological framework for interpreting plant‐associated microbiome differentiation in extensive green roof substrates.

## Graphical abstract

Three plant species selected from coal mine spoil heaps using a trait‐based approach were associated with distinct bacterial and fungal microbiome patterns in extensive green roof substrates. Discriminant taxa and differences in predicted metabolic pathways provided complementary taxonomic and functional perspectives on plant‐associated substrate microbiome differentiation.

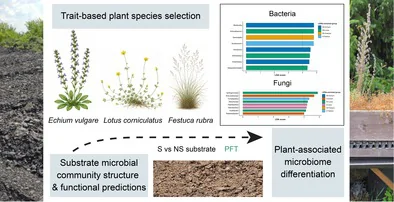

## 1 Introduction

Extensive green roofs (EGR) are environments with well‐defined characteristics, as determined by, among other things, their main components, i.e., the substrate and plants, which together form the basic functional layer of this system (Kader et al. 2022; Oberndorfer et al. 2007). Typically, EGR substrates are shallow (< 20 cm), lightweight growing media, composed of mixes of mineral components and aggregates, supporting low‐maintenance vegetation, and are therefore poor in organic matter and unstable in terms of moisture content (Ampim et al. 2010; Farah et al. 2024; Getter and Rowe 2006). Furthermore, the function of EGR substrates is largely dependent on biota, especially plant species with different ecological functions (Vannucchi et al. 2023). In addition, plants act as “ecosystem engineers” for green roofs (Seaborg 2022), influencing carbon sequestration, nutrient retention, and water retention regulation, while also altering the microclimate of the substrate (Steenaerts et al. 2026).

The composition of the substrate and the accompanying vegetation influence the core microbiota, creating the microbiological structure of the EGR (Larysz and Sierka 2024; McGuire et al. 2015; Van Dijck et al. 2024), which in turn influences the ecological functioning of the EGR ecosystems by, e.g., affecting plant stress tolerance and, potentially, productivity (Hoch et al. 2019; van der Heijden et al. 1998). However, most studies focus exclusively on plant composition (Heim and Lundholm 2022; Ndayambaje et al. 2024) or the microbiome in relation to dominated roof vegetation cover by *Sedum* spp., which is typically installed on EGR and characterized by lower microbial biodiversity (Farah et al. 2024; Hoch et al. 2019; Todeschini and Fett‐Neto 2025; Van Dijck et al. 2024), but do not analyse other, more diverse functional plant types, as highlighted by Fulthorpe et al. (2018).

The selected plant species, representing different functional groups, colonize the roof substrate and may modify substrate conditions through their physiological characteristics, reflected in functional traits (Lavorel and Garnier 2002), potentially contributing to differences in microbial niches. Sterilization of the substrate provided two starting points: one for the substrate with an existing community (non‐sterile variant), and one for the “biologically reset” substrate (sterile variant), which was the subject of the study, and provided two contrasting starting conditions for examining how plant species and substrate treatment are associated with microbial communities in the substrate, and whether plant‐associated conditions may contribute to differences in microbial community composition. Sterilization therefore provides a contrasting initial microbial condition in which differences in microbial communities developing in association with individual plant species can be examined, and plant functional traits were thus deemed essential for interpreting the taxonomic composition of microbial communities (Trivedi et al. 2020). We hypothesized that the three plant species would be associated with distinct patterns of microbial community composition in sterile and non‐sterile substrates.

Plant species were selected from coal mine spoil heaps using the habitat template approach (Lundholm 2006) and previous studies (Biela and Sierka 2023). Next‐generation sequencing was used to characterize bacterial and fungal communities and, together with selected functional traits of plants, to examine plant species‐specific patterns in microbial community composition and diversity in sterilized and non‐sterilized green roof substrates.

This study aimed to examine how three plant species representing different plant functional groups are associated with microbial communities in green roof substrates. Specifically, we sought to improve our understanding of interactions between plant species and soil microbial communities and to explore how species‐specific patterns in substrate‐associated microbial communities may contribute to the designing and management of more resilient and sustainable green roof ecosystems. To address this aim, we employed an integrated approach combining microbiome analysis (16S and ITS), analysis of plant functional traits, biomarker identification (LEfSe), co‐occurrence network analysis, and PICRUSt2 functional prediction. Such a broad combination of methods applied to extensive green roofs is relatively rare and constitutes the added value of this study.

## 2 Experimental Procedures

### 2.1 Materials and Methods

The experiment included two green roof substrate variants: sterilized (S) and non‐sterilized (NS). Each of them was sown separately with different plant seeds representing three main plant functional types: forb *Echium vulgare* L., legume *Lotus corniculatus* L., and grass *Festuca rubra* L., which made up the experimental variants, each in eight repetitions. The plant seeds, three for each species, were placed in plastic square pots (9 × 9 × 14 cm) in Saucer in the corresponding substrate type and left in the incubation chamber Fito700 BioGenet (20°C day/15°C night); photoperiod 15 h (day) and 9 h (night); humidity level at 50%; lighting parameters: 22% CW and 12% WW for seven weeks, and during that time they have been monitored and watered with 20 mL of tap water. The sterilized substrate variant was kept in a modified propagation greenhouse system with punctured PVC tubes attached to the lid for sterile irrigation. Sterilization of the substrate was carried out under controlled conditions in an ASVE series autoclave at 121°C temperature for 2 h, pressure 12 × 10^4^ Pa, following Wolf and Skipper (1994) guidelines. Non‐sterile substrates have not undergone any modifications. Sterilized substrates were used as controls for microbial assessments.

In this study, green roof substrate samples were collected from each repeated variant at three locations in proximity to the plant (±2 cm), taking care not to damage the root system. Substrate sampling was performed prior to the initiation of the experiment and repeated at its conclusion. The collected material was then transferred into sterile 2 mL Eppendorf tubes and subjected to molecular microbiome analyses.

### 2.2 Plant Seeds and Substrate

The seeds were obtained from the Institute of Plant Breeding and Acclimatization—National Research Institute (NIP‐PL: 5290007029): *Echium vulgare* L. (AN239070) (Ev), *Lotus corniculatus* L. (AN243282) (Lc), and *Festuca rubra* L. (AN236246) (Fr). The experiment was conducted under controlled laboratory conditions using the substrate for extensive green roof system compliant with the FLL (Forschungsgesellschaft Landschaftsentwicklung Landschaftsbau e.V.) Green Roofing Guidelines and the European Technical Assessment (ETA‐13/0557) (Extensive Multi‐Layer Substrate E‐light, ZIDA). The substrate used in the experiment is a manufactured lightweight green roof substrate composed mainly of expanded shale and clay, lava, pumice, crushed brick, Porlith, and green‐waste compost. According to the manufacturer's specification, the composition may vary by region.

Key plant functional traits relevant to microbiome modulation are summarized in Table 1.

**TABLE 1:** Functional traits of study plants and their expected influence on substrate microbiome assembly.

Plant species	Plant functional type (PFT)	Application rationale	Symbiotic potential	Expected microbial influence	Author/study
*Echium vulgare* L.	Forb	A monocarpic perennial, selected as a drought‐tolerant forb, typical for nutrient‐poor habitats; aided by a large root system, semi‐succulent leaves, and epidermal hairs on leaves	Low: no N‐fixing symbiosis	Microbial recruitment process dependent on substrate pH of root‐related microbes (e.g., *Rhizobium* sp., *Mycobacterium* sp., *Pseudomonas* sp., et al.)	Csorba et al. (2024); Klemow et al. (2002); Latini et al. (2022); Wala et al. (2021)
*Lotus corniculatus* L.	Legume	A perennial legume, adapted to poor quality soils and water‐deficient habitats; tolerates overwatering, drought, and soil salinity; its highly developed root system promotes symbiotic potential	High: rhizobial symbiosis and mycorrhizal associations	Increase in bacterial diversity in the rhizosphere area, influenced mainly by the root exudates	Borozan et al. (2020); Petrova et al. (2022); Sujkowska‐Rybkowska et al. (2020)
*Festuca rubra* L.	Grass	A perennial grass, representing a broad ecological amplitude; a stress‐tolerant and adjusted to low nutrient reserves in the soil, with a dense root system, influencing the mycorrhizal fungi association	Low: arbuscular mycorrhiza only	Restoring biological balance and strengthening the diversity and activity of microorganisms, especially under conditions of stress caused by pollution	Borowik et al. (2019); Corcoz et al. (2022); Kosová et al. (2022)

### 2.3 Substrate Measurements and Samples Preparation

At the conclusion of the experiment, substrate water content was measured once per repetition of the experimental variant. For this purpose, a WET‐2 sensor (Delta‐T Devices, Cambridge, UK) was used, with the sensor element placed as close as possible to the root zone of the plants (±3 cm), taking special care (Kargas et al. 2023).

Physicochemical analyses (pH, macronutrients (NO_3_^−^‐N, P, K, Ca, Mg, Cl) [mg·dm^−3^], salinity NaCl [g·dm^−3^], Na [mg·dm^−3^], organic matter [%], dry matter [%], and bulk density [kg·l^−3^]) were performed at the laboratory of the District Chemical‐Agricultural Station (Gliwice).

### 2.4 Plant Biomass

Fresh and dry biomass were measured for the aboveground part, i.e., the shoot including the stem and leaves, and the rhizosphere portion, i.e., the roots. Biomass was measured using a high‐precision Scientech Analytical Lab Electronic Balance SA120D model (Scientech Inc., CO, USA) with an accuracy of 0.001 g (Sun et al. 2024).

### 2.5 The Content of Pigments and Chlorophyll *a* Fluorescence

The pigment content was measured with a used Dualex Scientific + leaf clip (Force‐A, France) sensor for non‐destructive in situ measurement of chlorophyll content, flavonol index, and anthocyanin index in leaves (Cerovic et al. 2012).

Chlorophyll *a* fluorescence measurements were performed on the same leaves using Plant Efficiency Analyses (Pocket PEA fluorimeter, Hansatech Ltd., Pemtney, UK). Before analyses, each leaf was dark‐adapted for 30 min using leaf clips. For induction of fluorescence, red actinic light was used (wavelength at peak 650 nm; spectral line half‐width 22 nm) with the intensity of 3500 μmol·m^−2^·s^−1^, and 1 s of transient fluorescence was recorded (Goltsev et al. 2012; Kalaji et al. 2014; Tsimilli‐Michael and Strasser 2004; Stirbet et al. 2018). Leaf clips were applied to the upper leaf of each germinated plant (Ev, Lc) and the upper leaf of Fr grasses and left to adapt in the dark for 15 min before measurement, in accordance with the user manual. The fluorescence signal was collected with a maximum frequency of 105 points s^−1^ (every 10 μs) within 0–0.3 ms, after which the frequency of recording gradually decreased, collecting a total of 118 points within 1 s.

### 2.6 Photosynthesis and Gas Exchange Measurement

The LCpro‐SD instrument (ADC BioScientific Ltd.) was used to measure the intensity of photosynthesis and gas exchange and its selected parameters: photosynthetic rate (A) [μmol·m^−2^·s^−1^], transpiration rate [mmol·m^−2^·s^−1^], stomatal conductance to H_2_O (gs) [mol·m^−2^·s^−1^], and intercellular CO_2_ concentration (ci) [μmol·mol^−1^]. For this measurement, three upper leaves of each germinated plant (Ev, Lc, Fr) were selected and used for measurements in ten repetitions per experimental variant.

### 2.7 DNA Extraction and Data Collection

A total of 50 substrate samples were collected for molecular analyses. Two initial control samples were collected before the experiment, with one control sample representing each substrate variant. Following completion of the experiment, 48 experimental substrate samples were collected from the experimental units and submitted for molecular analyses. Thus, the dataset comprised 50 unique substrate samples in total: 2 initial controls and 48 experimental samples collected at the end of the experiment, which were then subsequently submitted to the designated Molecular Biology Techniques Laboratory (Adam Mickiewicz University, Poznan, Poland) to assess the finite response of substrate microbiota to substrate changes. Total genomic DNA was extracted from 200 mg of substrate. According to Child et al. (2024), the most optimal DNeasy PowerSoil Pro Kit (Qiagen, Germany) soil kit was used. Before PCR amplification, DNA extracts were normalized with sterile water to a concentration of 10 ng/μL. Blank DNA extraction was prepared and sequenced as a negative control.

For microbial community profiling, the hypervariable V4 region of the 16S rRNA gene (V4 16S rDNA) and internal transcribed spacer 1 (ITS1) were used. The V4 16S rDNA was amplified using 515F (GTGCCAGCMGCCGCGGTAA) (Lane et al. 1985) and 806R (GGACTACHVGGGTWTCTAAT) primer set (Caporaso et al. 2011). The ITS1 region was amplified using ITS1‐F‐KYO2 (TAGAGGAAGTAAAAGTCGTAA) and ITS2‐KYO2 (TTYRCTRCGTTCTTCATC) (Toju et al. 2012) primers. All PCR reactions were performed in two technical replicates, each containing Hot FIREPol DNA polymerase (Solis BioDyne), 0.25 μM of each primer tailed at the 5′‐ends with dual‐indexed Ion Torrent A and P adapters, and 1 μL of template DNA. The PCR reaction without a DNA template was used as a negative control. The amplification program was set as follows: 12 min at 95°C, followed by 35 cycles of 15 s at 95°C, 30 s at 50°C, and 30 s at 72°C, with a final extension step at 72°C for 5 min. Following amplification, for each PCR, 3 μL of DNA solution was electrophoresed on a 2% agarose gel to check amplification efficiency. Then, the amplicons were pooled and purified using a 2% E‐Gel SizeSelect II Agarose Gel System (Invitrogen), according to the manufacturer's protocol.

The DNA concentration and size distribution of the DNA libraries were determined using the High Sensitivity D1000 ScreenTape assay on the Agilent 2200 TapeStation System (Agilent Technologies). Clonal amplification of the template DNA was carried out with the Ion Torrent OneTouch System II and the Ion Torrent OT2 Kit, in accordance with the manufacturer's protocol. Sequencing was then performed using the Ion 540 Kit‐OT2 and the Ion S5 system on an Ion 540 chip (Thermo Fisher Scientific), generating single‐end reads of up to 500 bp. A minimum of 100,000 reads was planned per sample.

### 2.8 Molecular Analysis

Initial filtering of raw sequence data was performed using Ion Torrent Suite software version 5.18.1 (Life Technologies, USA) to eliminate polyclonal and low‐quality reads. Subsequent bioinformatic processing utilized FASTQ files within a custom workflow. Reads shorter than 200 bp were excluded using Geneious Prime 2025.0.5 (Biomatters Ltd.). The FastX‐Toolkit (Hannon 2010) was employed to retain only sequences with at least 50% of bases having a quality score of 25 or higher. Quality‐filtered reads were then demultiplexed based on index combinations using Geneious Prime. PCR primers were trimmed from both 5′ and 3′ ends. Amplicon Sequence variants (ASVs) were denoised with the DADA2 denoise‐pyro algorithm implemented in QIIME2 version 2025.4 (Bolyen et al. 2019; Callahan et al. 2016). Reads were quality‐filtered using maxEE = 2 and truncQ = 2, and chimeric sequences were identified and removed using the consensus method. ASVs detected in control samples were removed using the UNCROSS2 algorithm (Edgar 2018). Prokaryotic ASVs were taxonomically assigned using the SILVA SSU rRNA database version 138.1 [www.arb‐silva.de] (Glöckner et al. 2017; Quast et al. 2013; Yilmaz et al. 2014). Fungal ASVs targeting the ITS1 region were extracted with ITSx version 1.1.3 (Bengtsson‐Palme et al. 2013) and subsequently assigned taxonomy using the UNITE database version 10.0 (Abarenkov et al. 2023).

### 2.9 Functional Prediction of Microbial Communities

The functional potential of prokaryotic communities was inferred using the Phylogenetic Investigation of Communities by Reconstruction of Unobserved States (PICRUSt2, v2.4.1) software package (Douglas et al. 2020). Amplicon sequence variant (ASV) tables generated from 16S rRNA gene sequencing data were used to predict metagenomic functional profiles, including Kyoto Encyclopedia of Genes and Genomes (KEGG) Orthologs (KOs) (Kanehisa 2019; Yi et al. 2020; Kanehisa et al. 2021). Predicted gene family abundances were subsequently used to infer metabolic pathways, which were classified according to the MetaCyc pathway hierarchy. The Nearest Sequenced Taxon Index (NSTI) was calculated for each sample and used as an indicator of the reliability of functional predictions (Langille et al. 2013). Based on their potential relevance to plant physiology, ten MetaCyc pathways were selected for subsequent statistical analysis.

### 2.10 Statistical Analysis

Statistical analysis of plant functional trait measurements was performed using Statistica version 13.3. The nonparametric Mann–Whitney U test was used to assess the presence or absence of statistically significant differences in parameters between the three plant species in the two media variants. Microbiome diversity was assessed using the Shannon‐Wiener (H′) indices (Revelle 2025; R Core Team 2022).

The comparison of microbial ASV richness with relative abundance of > 0,1% was conducted in this research. Co‐occurrence networks were inferred using the SparCC algorithm to account for the compositional nature of relative‐abundance microbiome data. Statistical significance of the inferred correlations was assessed using 100 bootstrap iterations to generate pseudo‐*p* values. Only correlations with pseudo‐*p* < 0.05 were retained for network construction. Each network was inferred independently for each experimental group using eight biological replicates (*n* = 8 per group). The resulting networks were visualized in Gephi (version 0.10.1) using the Fruchterman–Reingold layout with the area parameter set to 10,000 and the gravity parameter set to 10. The associations between bacteria and fungi were represented as ecological networks, where each taxon served as a node and its interactions were depicted as links. These networks were constructed using a Spearman correlation matrix, using the ‘psych’ package version 2.5.6 in R version 4.3.3 (Revelle 2025; R Core Team 2022). Alpha‐rarefaction curves and diversity analyses were performed in QIIME2. Rarefaction curves were generated using the Shannon diversity index to evaluate sampling completeness and determine whether sequencing depth was sufficient to capture microbial diversity within samples. Shannon diversity index was compared among experimental groups using the Kruskal–Wallis test followed by pairwise comparisons with Benjamini–Hochberg correction for multiple testing. Beta diversity was evaluated using Bray–Curtis dissimilarity matrices derived from ASV abundance data and visualized through principal coordinates analysis. Indicator taxa associated with individual groups were identified using the indicspecies package in R. The analysis was performed with the multipatt function using the IndVal.g index. Statistical significance was assessed using a permutation test with 9999 permutations. The resulting *p*‐values were adjusted for multiple testing using the Benjamini–Hochberg correction. Community composition was compared using permutational multivariate analysis of variance based on Bray–Curtis dissimilarities with the adonis2 function from the vegan package in R. The effects of soil sterilization, plant species, and their interaction were evaluated using 9999 permutations. Marginal PERMANOVA was additionally performed to compare the variation explained by soil sterilization and plant species. Homogeneity of multivariate dispersion was assessed using the betadisper function with 9999 permutations.

Statistical analysis of predicted functional pathways included six experimental groups defined by plant species and substrate sterilization effect. Control samples were excluded from the factorial analysis. For each of the ten selected PICRUSt2‐predicted pathways, a two‐way general linear model (GLM) was fitted with plant species, substrate sterilization, and their interaction (species × sterilization) as fixed factors. The significance of the main effects and their interaction was assessed using analysis of variance (ANOVA) based on the fitted GLM (Hector et al. 2010). Estimated marginal means (EMMs) were subsequently calculated for each species × sterilization combination to characterize the direction and magnitude of treatment effects (Garofalo et al. 2022). When significant effects were detected, pairwise comparisons were performed using Tukey's honestly significant difference (HSD) test, with *p*‐values adjusted for multiple comparisons. In particular, comparisons between non‐sterilized and sterilized substrates were examined within each plant species. Statistical significance was defined as *p* < 0.05.

### 2.11 Discriminant Taxa Analysis

Differential abundance analysis was performed to identify microbial taxa associated with experimental groups. Taxonomic abundance profiles were summarized at the genus level and analysed using the Linear Discriminant Analysis Effect Size (LEfSe) approach, which integrates non‐parametric statistical testing with linear discriminant analysis to identify differentially abundant microbial biomarkers (Segata et al. 2011).

LEfSe analysis was performed in R version 4.6.1 using the microbiomeMarker package version 1.13.2 (Cao et al. 2022). The Kruskal–Wallis rank‐sum test was applied to identify taxa showing significant differences among experimental groups, followed by linear discriminant analysis (LDA) to estimate effect sizes. Taxa with Kruskal–Wallis *p*‐values ≤ 0.05 and LDA scores ≥ 2 were considered discriminant biomarkers. Taxa with LDA scores ≥ 4 were classified as strong discriminant biomarkers (Van Dijck et al. 2024).

Relative abundance values of identified discriminant taxa were extracted from genus‐level abundance matrices. Pairwise comparisons were additionally performed using the Wilcoxon rank‐sum test to evaluate enrichment patterns of identified biomarkers.

## 3 Results

### 3.1 Substrate Physicochemical Analysis

Physicochemical properties of the experimental substrate were determined after completion of the plant‐growth experiment and therefore characterized the substrate following the experimental period and were used to document the experimental conditions. Because substrate physicochemical measurements were available as single measurements for each experimental variant, these data were evaluated descriptively, and no statistical comparison among variants was performed. Across the six experimental variants, pH ranged from 7.8 to 8.0, P from 86 to 101 mg·dm^−3^, K from 101 to 113 mg·dm^−3^, Ca from 1497 to 1663 mg·dm^−3^, Mg from 266 to 291 mg·dm^−3^, Na from 34 to 39 mg·dm^−3^, organic matter from 2.26% to 2.67%, dry matter from 98.73% to 99.15%, and bulk density from 1.209 to 1.291 kg·L^−1^. NO_3_‐N concentrations ranged from 0 to 13 mg·dm^−3^, whereas chloride was reported as 0 mg·dm^−3^ in all experimental variants. NaCl was below the analytical detection limit according to the laboratory analysis and thus reported as 0 in five variants, and only detected as 0.14 g·dm^−3^ in the NS‐Fr variant.

The measured physicochemical properties differed from some of the manufacturer's specifications, which characterize the commercial substrate prior to its use in the experiment (Table S1). Such differences are expected because the experimental substrate was analysed after the plant‐growth period and therefore represents the physicochemical state of the substrate following the experimental treatments.

### 3.2 Plant Functional Traits. Biomass

Mean plant biomass responses to substrate type varied among species (Figure 1). Ev revealed higher shoot fresh biomass in sterilized substrate (NS 0.642 g vs. S 0.839 g; Figure 1A), whereas shoot dry biomass was significantly higher in non‐sterilized substrate (NS 0.080 g vs. S 0.050 g) (*p* = 0.04; Figure 1D). Root fresh biomass was slightly higher in sterilized substrate (NS 0.383 g vs. S 0.463 g; Figure 1G), whereas root dry biomass was slightly higher in non‐sterilized substrate (NS 0.014 g vs. S 0.012 g; Figure 1J); neither difference was significant. For Lc, shoot fresh (NS 0.595 g vs. S 0.441 g) and dry (NS 0.089 g vs. S 0.062 g) biomass were higher in non‐sterilized substrate (Figure 1B,E), as was rhizosphere fresh (NS 0.454 g vs. S 0.388 g) and dry (NS 0.027 g vs. S 0.013 g) biomass (Figure 1H,K). In Fr, shoot dry biomass was significantly higher in sterilized substrate (NS 0.012 g vs. S 0.215 g; *p* = 0.03; Figure 1F), but was not evident for fresh shoot biomass (NS 0.758 g vs. S 0.629 g; Figure 1C). The same observation was made for root dry biomass (NS 0.004 g vs. S 0.034 g; Figure 1L) and fresh root biomass (NS 0.400 g vs. S 0.202 g; Figure 1I), although neither difference was significant. Overall, substrate type influenced biomass allocation differently across species, with no general significant difference in fresh biomass, and only in dry biomass.

**FIGURE 1:**
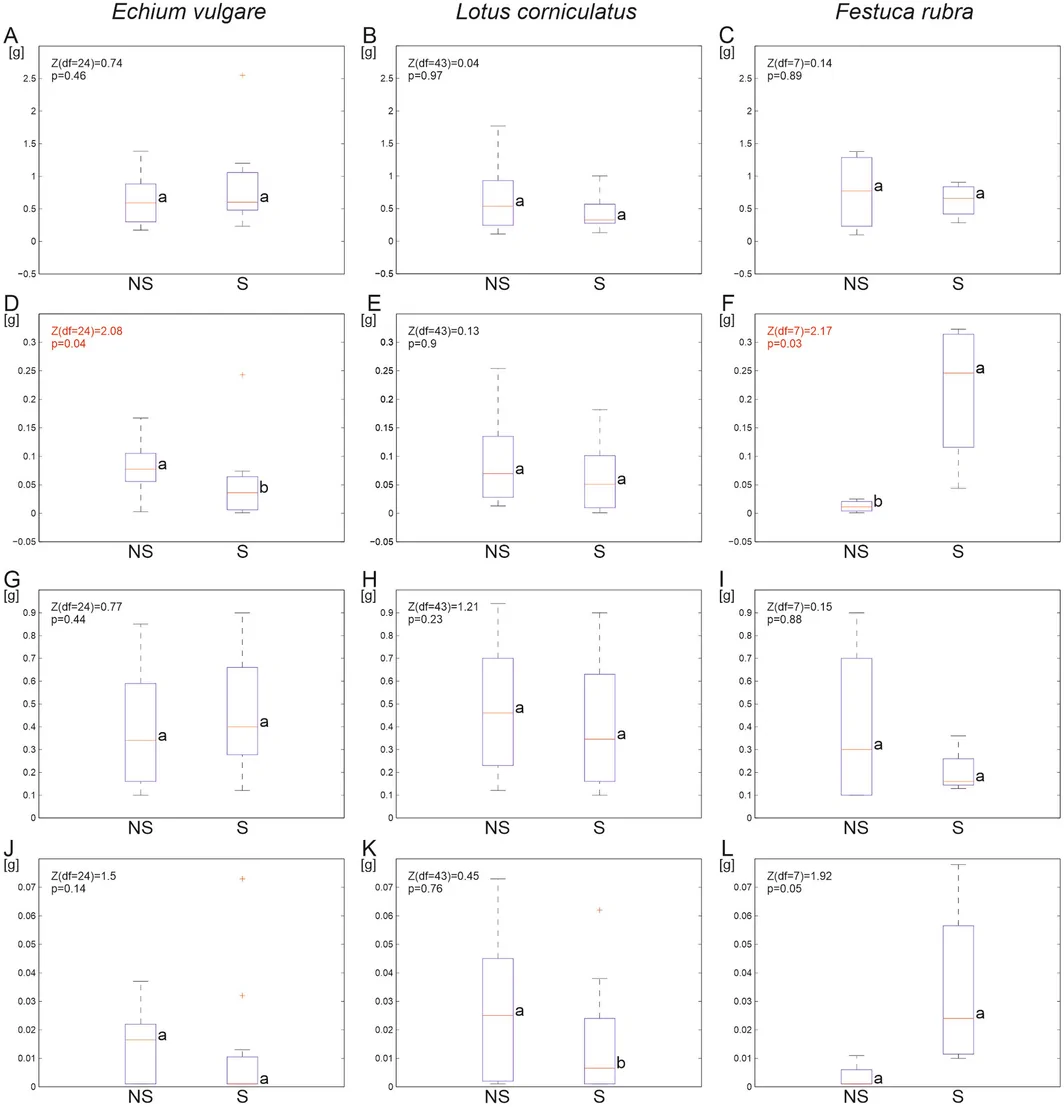
Biomass of the aboveground plant body for NS and S variants: fresh (A–C) and dry (D–F) for Ev (A, D), Lc (B, E), and Fr (C, F), and underground plant body for NS and S variants: fresh (G–I) and dry (J–L) for Ev (G, J), Lc (H, K), and Fr (I, L). The graphs show the Mann–Whitney *U* statistic (Z), the number of degrees of freedom (df), and the *p*‐value. Statistically significant differences were marked in red. The difference letters a and b indicate the differences.

### 3.3 Plant Functional Traits. Photosynthetic Performance and Substrate Water Content Availability

Fluorescence readings suggest that Ev is the least sensitive to change in substrate conditions, as parameters remain stable, with the sole exception of fluorescence area (*p* < 0.05). For Lc, an increase in F_o_ and F_m_ compared to the variant S may indicate an increase in photosynthetic activity or oxidative stress (*p* < 0.05). Fr grass exhibited mostly disadvantageous responses for non‐sterile substrate and was preferable for sterile, as a decrease in most parameters in non‐sterilized substrate may point to unfavourable conditions, associated with a decrease in photosynthetic activity (*p* < 0.05) (Figure 2A).

**FIGURE 2:**
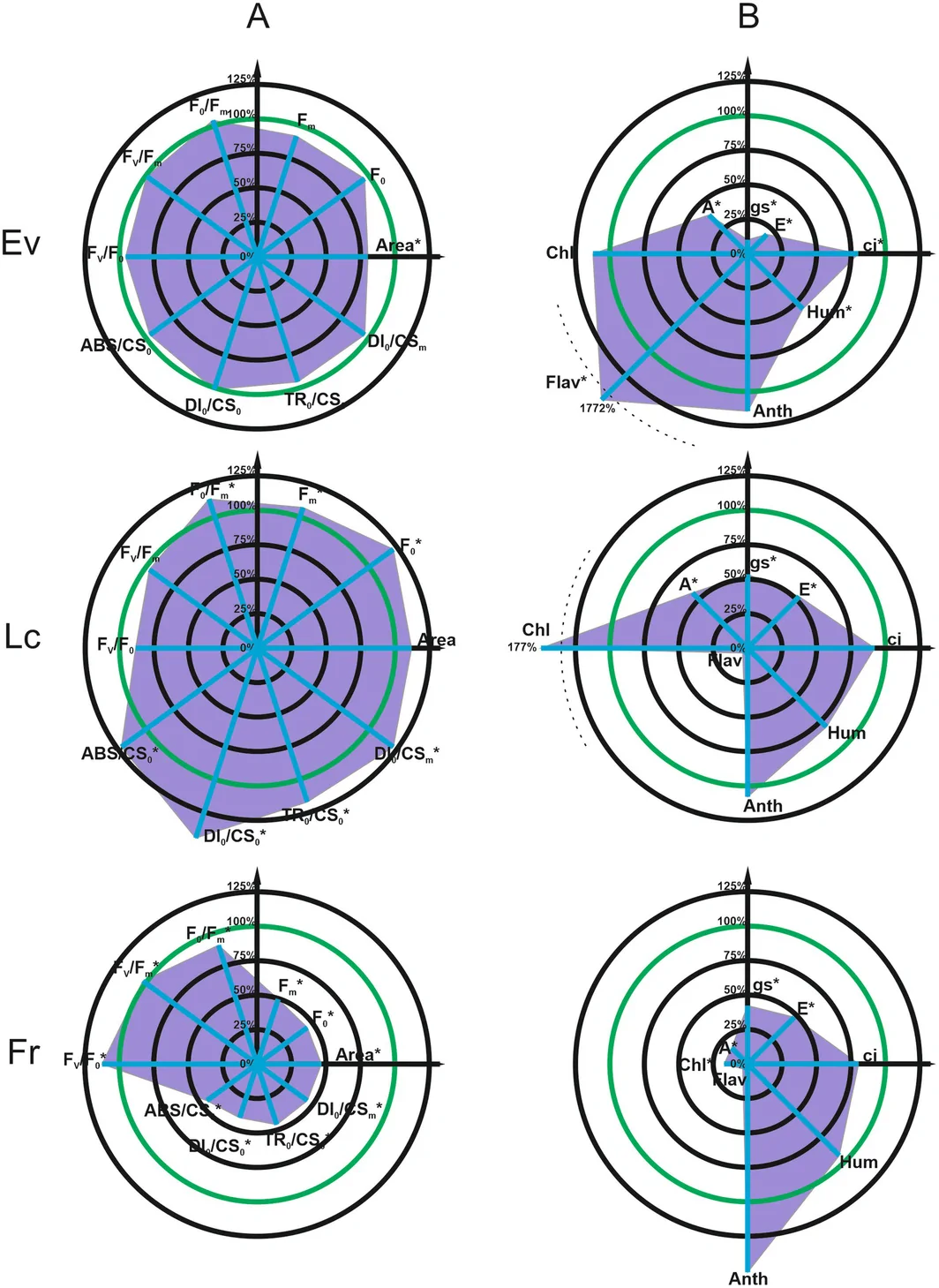
Radar charts of percentage fluorescence parameters of study plants (A), and radar charts of percentage pigment content, chlorophyll *a* fluorescence, gas exchange, and substrate moisture of experimental variants (B). The parameters that were measured for the sterilized substrate were arbitrarily set to 100%. Accordingly, each parameter value is shown as a relative value to the sterilized substrate and marked in the purple area. Values of the parameters for the sterilized substrate variants were taken as 100% and marked with a green circle on the graphs. Statistically significant differences (*p* < 0.05) in parameters are marked with an asterisk.

The parameters of the photosynthetic apparatus efficiency of plants in NS show lower efficiency compared to those in the S substrate for all three plant species (Figure 2B). Pigments and those affecting photosynthesis are higher in Ev plants, with significant statistical differences found for all gas exchange parameters, flavonol content, and substrate moisture content (*p* < 0.01). In Lc plants, the amount of chlorophyll *a* increased significantly compared to the sterile substrate, and the stress level decreased by almost 100%, which should translate into the efficiency of the photosynthetic apparatus. At the same time, the anthocyanin content is nearly 10% higher than in the sterile substrate. Significant differences were observed only for selected respiration parameters: E and gs (*p* < 0.01). Fr grass showed significantly reduced photosynthetic efficiency in non‐sterilized substrate (E, gs, A: *p* < 0.01). Chlorophyll *a* content was low, less than 25% of the value for plants grown in sterilized substrate (*p* < 0.05). The only parameter exceeding S values was anthocyanin content, exhibiting a common pattern for all three plants. Fluorescence and pigment analyses indicate that Ev is the least sensitive to substrate changes, Lc shows increased chlorophyll and reduced stress in non‐sterile substrate, while Fr experiences strongly reduced photosynthetic efficiency, with anthocyanin content elevated in all three plant species in non‐sterile substrates.

### 3.4 Sequencing Output and Data Quality

A total of 5,184,779 raw reads were generated for the V4 region of the 16S rRNA gene and 4,049,270 raw reads for the ITS marker. Following quality filtering, 4,671,637 high‐quality V4 16S rRNA gene reads were retained for downstream analyses. On average, 93,432 reads were obtained per sample (median: 96,138 reads). After denoising, a total of 730 ASVs were identified for the V4 16S rRNA dataset. Prior to downstream diversity and community composition analyses, samples were rarefied to an even sequencing depth of 50,000 reads per sample. For the ITS dataset, quality filtering and removal of non‐target eukaryotic sequences resulted in 609,801 high‐quality reads. The mean sequencing depth was 12,196 reads per sample (median: 11,511 reads). Denoising yielded 268 ITS ASVs. Prior to downstream analyses, ITS samples were rarefied to 5000 reads per sample to account for differences in sequencing depth among samples.

Rarefaction curves for bacterial and fungal communities approached saturation in all samples, indicating that sequencing depth was sufficient to capture the majority of microbial diversity (Figures S1 and S2).

Weighted NSTI values ranged from 0.006 to 0.077 (mean ± SD = 0.047 ± 0.014), indicating that the microbial communities analysed were generally well represented by genomes available in reference databases.

### 3.5 Alpha Diversity

Bacterial alpha diversity differed significantly between non‐sterilized and sterilized soil. Pairwise comparisons showed that all non‐sterilized groups (Ev, Fr, and Lc) differed significantly from all sterilized groups (adjusted *q* = 0.00396–0.00507). In contrast, no significant differences in bacterial alpha diversity were detected among plant species within either the non‐sterilized or sterilized soil (all adjusted *q* > 0.05) (Figure S3). Fungal alpha diversity did not differ significantly among any of the experimental groups after correction for multiple comparisons (Figure S4).

### 3.6 Microbial Community Composition

Microbial community composition was assessed using eight biological replicates per experimental group (*n* = 8). The no‐plant control was sampled at the before timepoint, whereas all plant‐associated samples shown in Figures 3 and 4 were collected at the after timepoint. Thus, Figures 3 and 4 present community composition following the experimental period, while the no‐plant control provides a baseline characterization of the substrate microbiome before plant establishment.

**FIGURE 3:**
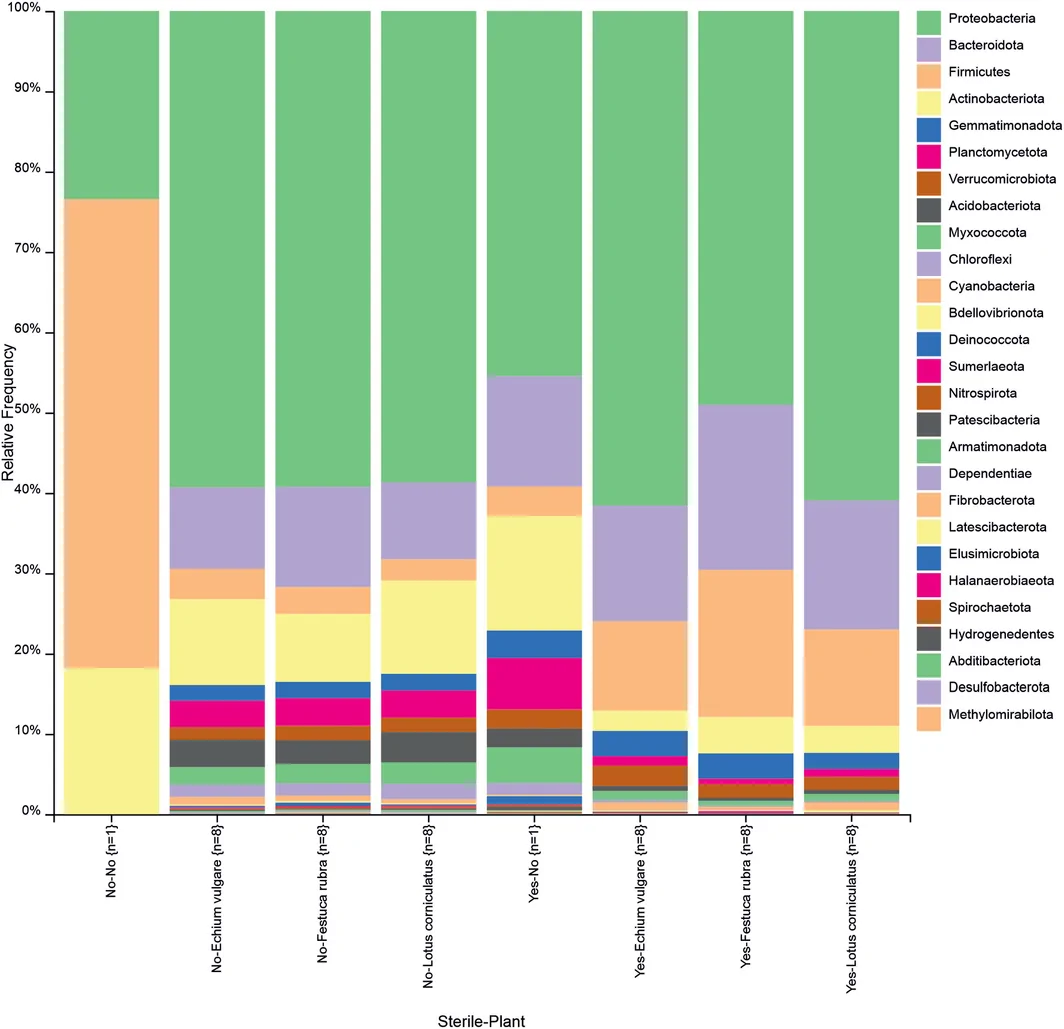
Relative abundance of bacterial taxa across experimental groups. Each plant–substrate treatment is represented by eight biological replicates (*n* = 8). Plant‐associated samples were collected at the after timepoint, whereas the no‐plant control was collected at the before timepoint.

**FIGURE 4:**
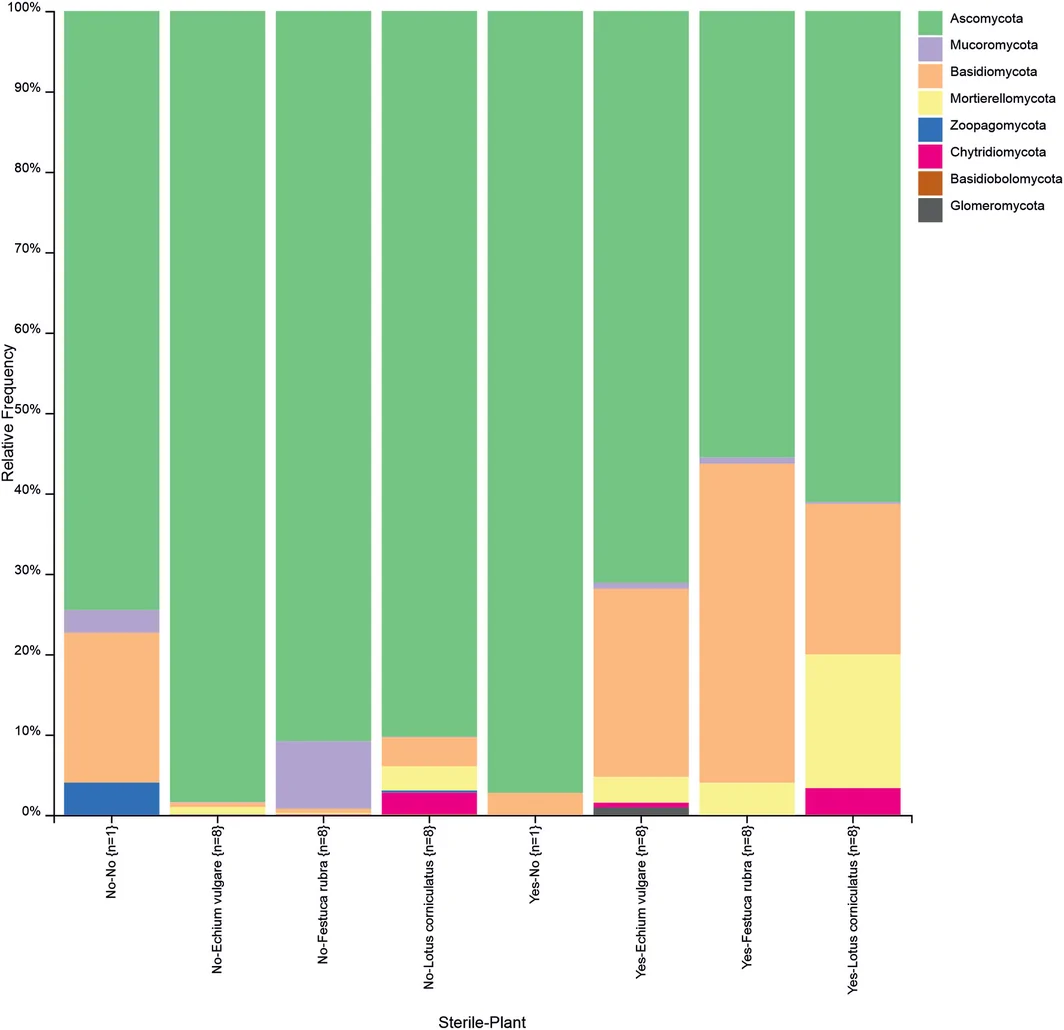
Relative abundance of fungal taxa across experimental groups. Each plant–substrate treatment is represented by eight biological replicates (*n* = 8). Plant‐associated samples were collected at the after timepoint, whereas the no‐plant control was collected at the before timepoint.

*Proteobacteria, Bacteroidota, Firmicutes*, and *Actinobacteriota* (Figure 3), as well as *Basidiomycota* and *Ascomycota* (Figure 4), dominate both NS and S variants, Shannon‐Wiener index range for bacteria 6.38–6.43 (NS), 5.71–5.89 (S), and fungi 2.18–4.45 (NS), 3.90–5.17 (S). The results also revealed that Lc plant demonstrates high overall species richness and evenness within both substrate variants: H′ for bacteria 6.38 (NS), 5.71 (S), and fungi 4.45 (NS), 4.92 (S). The lowest in fungal richness across all experimental variants was Fr grass: H′ for fungi 3.21 (NS), 3.90 (S), bacteria 6.43 (NS), 5.75 (S). Ev, on the other hand, was the richest in fungal diversity, H′ was equal to 2.18 (NS), 5.17 (S) for fungi, and 6.38 (NS), 5.89 (S) for bacteria.

### 3.7 Differential Abundance of Microbial Taxa

Indicator species analysis identified 42 significant indicator taxa (Table 2). Overall, the majority of indicator taxa were associated with plants grown in non‐sterilized substrate (33 taxa), including Lc (19 taxa), Fr (9 taxa), and Ev (5 taxa). In contrast, only nine indicator taxa were associated with plants grown in sterilized substrate, comprising four taxa each for Fr and Lc and a single taxon for Ev. The strongest indicator taxa were *Actinotalea* for Lc (IndVal.g = 0.935), *Herminiimonas* for Fr (IndVal.g = 0.890), and *Pseudomonadaceae* for Ev (IndVal.g = 0.910) in the non‐sterilized substrate, whereas *Marmoricola* (IndVal.g = 0.734), *Paracoccus* (IndVal.g = 0.741), and *Methylobacterium–Methylorubrum* (IndVal.g = 0.823) showed the highest indicator values for Lc, Fr, and Ev, respectively, in the sterilized substrate. In contrast, no significant fungal indicator taxa were identified after correction for multiple testing.

**TABLE 2:** Indicator taxa associated with the experimental groups.

Taxon	Indicator group	IndVal.g	*p*	*p*‐adjusted BH
*Kribbella*	NS‐Ev	0.8084132132	0.0001	0.0028666667
***Pseudomonadaceae***	NS‐Ev	0.9095071137	0.0003	0.0045866667
*Nonomuraea*	NS‐Ev	0.7745511234	0.0003	0.0045866667
*Nitrosospira*	NS‐Ev	0.7065197851	0.0004	0.0045866667
*Pseudonocardia*	NS‐Ev	0.7198685795	0.0008	0.0071172414
*Hydrogenedensaceae*	NS‐Fr	0.7512959168	0.0001	0.0028666667
*Algoriphagus*	NS‐Fr	0.7905694150	0.0002	0.0038222222
*Limnobacter*	NS‐Fr	0.8502095500	0.0004	0.0045866667
*Gulbenkiania*	NS‐Fr	0.7905694150	0.0003	0.0045866667
*Erwiniaceae*	NS‐Fr	0.7652177878	0.0006	0.0057333333
*Armatimonadales*	NS‐Fr	0.7071067812	0.0018	0.0103200000
*Spirosomaceae*	NS‐Fr	0.7071067812	0.0021	0.0112875000
*Methyloversatilis*	NS‐Fr	0.7348598480	0.0022	0.0115836735
*Truepera*	NS‐Fr	0.7164322024	0.0101	0.0323701863
***Actinotalea***	NS‐Lc	0.9354143467	0.0001	0.0028666667
*Arthrobacter*	NS‐Lc	0.8660254038	0.0001	0.0028666667
*Candidatus collierbacteria*	NS‐Lc	0.8660254038	0.0001	0.0028666667
*Inquilinus*	NS‐Lc	0.8660254038	0.0001	0.0028666667
***Herminiimonas***	NS‐Lc	0.8660254038	0.0001	0.0028666667
*Solibacillus*	NS‐Lc	0.8204696316	0.0001	0.0028666667
*Elsterales*	NS‐Lc	0.8052329827	0.0001	0.0028666667
*Tahibacter*	NS‐Lc	0.7718264476	0.0001	0.0028666667
*Vermiphilaceae*	NS‐Lc	0.7246733039	0.0002	0.0038222222
*Emticicia*	NS‐Lc	0.7926209327	0.0004	0.0045866667
*Rhodothermaceae*	NS‐Lc	0.7674658384	0.0004	0.0045866667
*Salinispora*	NS‐Lc	0.7278717625	0.0004	0.0045866667
*Candidatus solibacter*	NS‐Lc	0.7068140936	0.0003	0.0045866667
*Paracaedibacteraceae*	NS‐Lc	0.7044539967	0.0004	0.0045866667
*Phycisphaerae*	NS‐Lc	0.7049976167	0.0005	0.0053750000
***Marmoricola***	NS‐Lc	0.7009260298	0.0006	0.0057333333
***Paracoccus***	NS‐Lc	0.7195931889	0.0010	0.0078181818
*Terrabacter*	NS‐Lc	0.7071067812	0.0023	0.0118680000
*Limnochorda*	NS‐Lc	0.7071067812	0.0023	0.0118680000
***Methylobacterium‐Methylorubrum***	S‐Ev	0.7155238264	0.0053	0.0205624060
*Symbiobacteraceae*	S‐Fr	0.7318857125	0.0004	0.0045866667
*Flaviaesturariibacter*	S‐Fr	0.7071067812	0.0019	0.0107736264
*Phaselicystis*	S‐Fr	0.7394260734	0.0027	0.0127816514
*Streptococcus*	S‐Fr	0.7456545143	0.0108	0.0336790419
*Anaerosalibacter*	S‐Lc	0.7905694150	0.0002	0.0038222222
*Alsobacter*	S‐Lc	0.7147097027	0.0006	0.0057333333
*Blastomonas*	S‐Lc	0.7248760788	0.0028	0.0130162162
*Brevibacillus*	S‐Lc	0.8256149699	0.0072	0.0251027027

*Note:* Bold indicates the microbial taxons.

### 3.8 Community Structure

PERMANOVA revealed that soil sterilization significantly affected bacterial community composition, explaining 36.6% of the total variation (*R* ^2^ = 0.366, pseudo‐*F* = 27.45, *p* = 0.0001). In contrast, plant species had no significant effect (*R* ^2^ = 0.034, pseudo‐*F* = 1.29, *p* = 0.211), and the interaction between sterilization and plant species was also non‐significant (*R* ^2^ = 0.039, *p* = 0.136). Marginal PERMANOVA confirmed that soil sterilization explained substantially more variation than plant species (*R* ^2^ = 0.366 vs. 0.034).

Soil sterilization also significantly affected fungal community composition, although its effect was considerably weaker (*R* ^2^ = 0.063, pseudo‐*F* = 2.93, *p* = 0.0006). Neither plant species (*R* ^2^ = 0.030, *p* = 0.938) nor the interaction between plant species and sterilization (*R* ^2^ = 0.028, *p* = 0.970) significantly influenced fungal community composition.

PCoA revealed distinct patterns of bacterial and fungal community composition, with stronger separation among bacterial samples and greater dispersion of fungal samples (Figure 5A,B).

**FIGURE 5:**
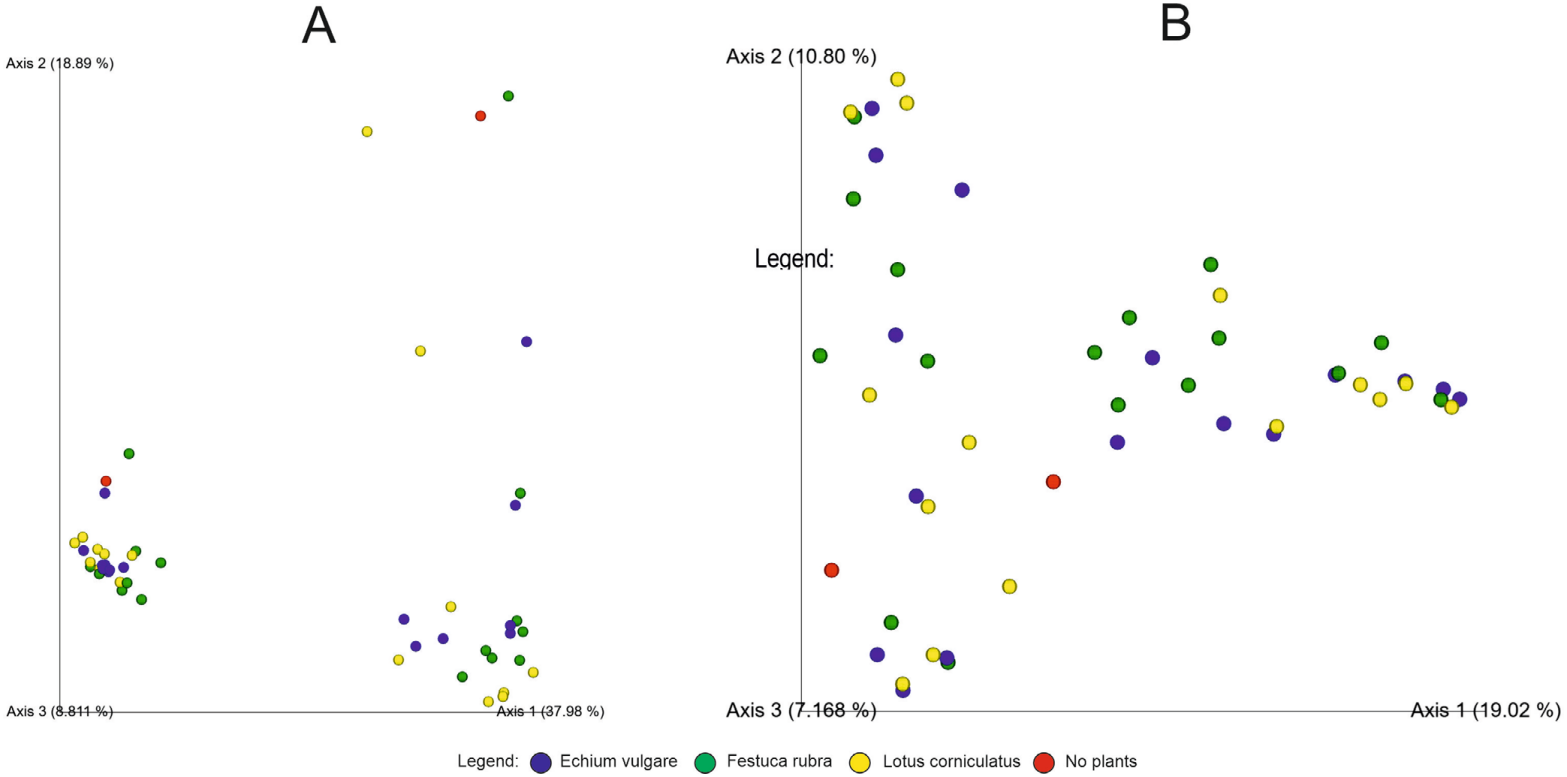
Principal coordinates analysis (PCoA) based on Bray–Curtis dissimilarities of bacterial (A) and fungal (B) communities across experimental groups.

### 3.9 Ecological Network Analysis

To characterize potential associations between bacterial and fungal communities across the studied substrate variants, microbial co‐occurrence networks were constructed (Figure 6). For each combination of plant species and substrate variant, eight biological replicates were available; these were used to calculate a correlation matrix using the SparCC method, on the basis of which a single co‐occurrence network representing the given experimental group was constructed. The correlation results and statistical values are presented in Dataset S1.

**FIGURE 6:**
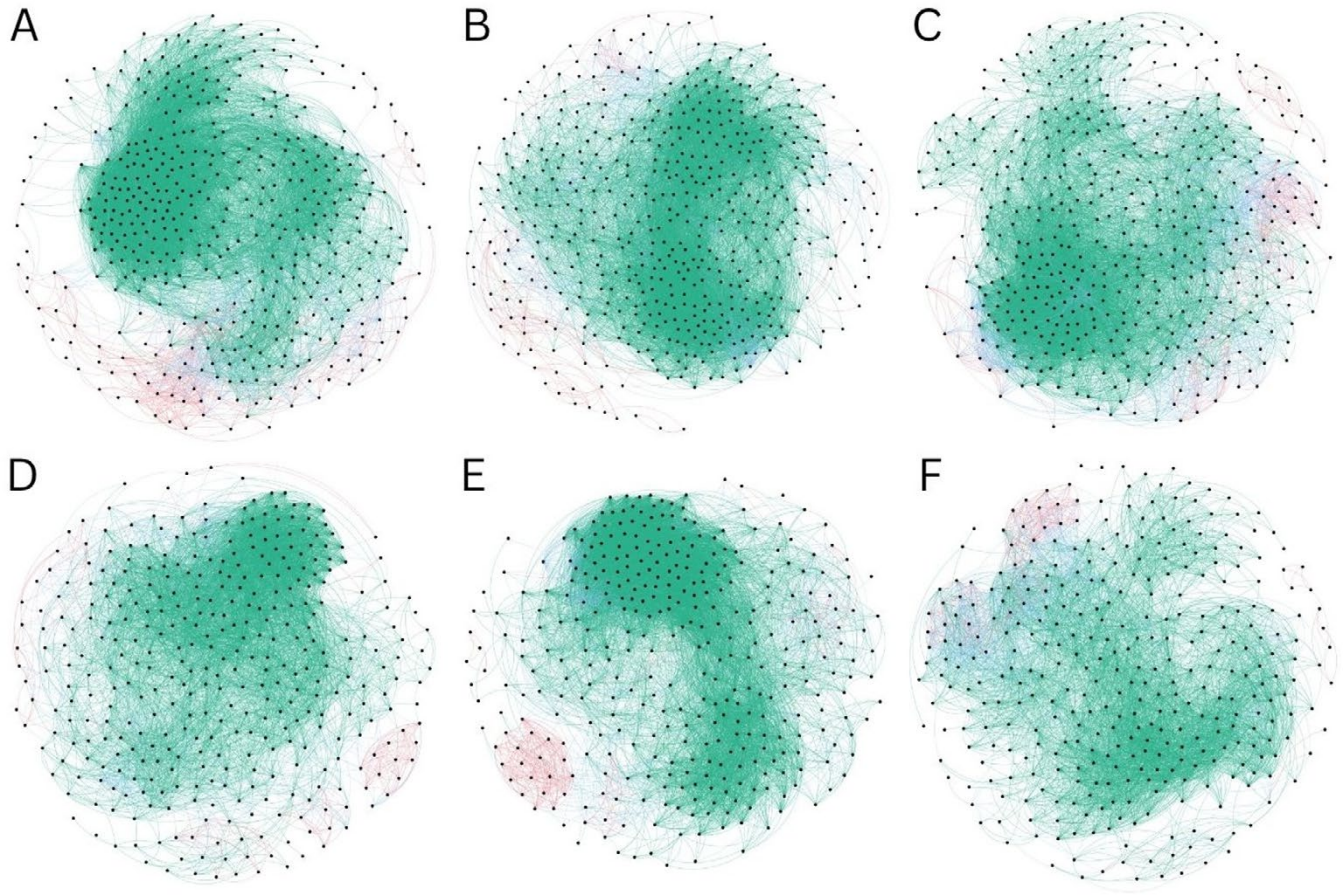
Ecological networks of substrate microorganisms for studied substrate variants: NS (A–C: Ev, Lc, Fr) and S (D–F: Ev, Lc, Fr). Edge colours denote interaction type: green, bacterial–bacterial; red, fungal–fungal; blue, bacterial–fungal.

The microbial co‐occurrence networks differed in their overall complexity and interaction patterns among the experimental variants, especially between non‐sterilized (NS) and sterilized (S) substrate variants (Table S2). The network analysis revealed networks comprising 353–509 nodes and 6.125–13.715 edges, with an average degree ranging from 34.70 to 61.23, density from 0.098 to 0.162, and modularity from 0.380 to 0.466 across the studied variants. The co‐occurrence networks were mainly dominated by bacteria‐bacteria interactions (B‐B), which accounted for 84.03%–93.72% of all network edges across the studied variants, whereas fungal‐fungal interactions (F‐F) averaged 2.48% of all network edges. The predominance of bacterial associations was accompanied by a variable balance between positive and negative correlations across the studied variants, with positive interactions most pronounced in the NS‐Ev (78.8% of all edges; Figure 6A) and S‐Ev variants (59.6% of all edges; Figure 6D), and negative interactions increasing in the remaining variants and reaching 48.9% in S‐Lc (Figure 6E) and 47,2% in NS‐Lc (Figure 6B). Notably, bacterial–fungal associations were relatively more frequent in the Fr networks, accounting for 10.3% and 12.7% of edges in NS‐Fr (Figure 6C) and S‐Fr (Figure 6F), respectively.

### 3.10 Identification of Discriminant Microbial Taxa

LEfSe analysis performed at the genus level identified 65 bacterial and 13 fungal discriminant taxa associated with the analysed experimental groups (Tables S3 and S4). Among these, 8 fungal and 7 bacterial taxa showed strong discriminatory potential (LDA ≥ 4), whereas 5 fungal and 58 bacterial taxa displayed moderate discriminatory effects (2 ≤ LDA < 4).

For bacterial communities, seven genera were identified as strong discriminant biomarkers based on V4 profiles. The strongest bacterial discriminator was *Sphingomonas* (LDA = 4.89), associated with the NS‐Lc variant, followed by *Brevundimonas* (LDA = 4.66; S‐Ev), *Tumebacillus* (LDA = 4.33; S‐Fr), and *Arenimonas* (LDA = 4.24; NS‐Lc). Additional strong bacterial biomarkers included *Paenibacillus* (LDA = 4.19; S‐Lc), *Brevibacillus* (LDA = 4.16; S‐Lc), and *Acidibacter* (LDA = 4.12; NS‐Lc) (Figure S5). The ITS‐based analysis revealed eight fungal genera classified as strong discriminant biomarkers. The highest discriminatory effects were observed for *Mortierella* (LDA = 4.81), *Schizothecium* (LDA = 4.80), *Serendipita* (LDA = 4.80), and *Acremonium* (LDA = 4.79). These taxa were associated with distinct experimental groups, including NS‐Ev (*Mortierella*), NS‐Lc (*Schizothecium*), NS‐Fr (*Serendipita*), and S‐Fr (*Acremonium*). Other strongly discriminating fungal genera included *Ascobolus* (LDA = 4.56), *Arthrobotrys* (LDA = 4.51), *Arachniotus* (LDA = 4.48), and *Neopyrenochaeta* (LDA = 4.35), enriched mainly in NS‐Ev and NS‐Lc variants (Figure S6).

In addition to strong biomarkers, LEfSe identified five fungal genera with moderate discriminatory strength. These included *Petriella* (LDA = 3.85; NS‐Fr), *Mycothermus* (LDA = 3.67; NS‐Lc), *Basidiobolus* (LDA = 3.62; NS‐Lc), *Exophiala* (LDA = 3.46; NS‐Ev), and *Mycofalcella* (LDA = 3.31; NS‐Lc). The bacterial dataset contained a larger number of moderate discriminant taxa, with 58 genera showing LDA scores between 2 and 4. The highest‐ranking taxa within this category included *Roseisolibacter* (LDA = 3.99; S‐Ev), *Caulobacter* (LDA = 3.94; S‐Ev), *Chryseolinea* (LDA = 3.92; NS‐Fr), *Cohnella* (LDA = 3.90; S‐Lc), and *Pseudoxanthomonas* (LDA = 3.87; NS‐Fr). Additional bacterial genera with relatively high discriminatory effects included *Haliangium*, *Aquicella*, *Pirellula*, *Hydrogenophaga*, and *Ohtaekwangia* (LDA range: 3.61–3.74).

### 3.11 Functional Prediction

PICRUSt2‐based functional profiling identified 517 microbial pathways, 10 of which had potential relevance to plant physiology and were selected for further functional prediction analysis (Tables S5 and S6). Two‐way ANOVA revealed a significant effect of substrate sterilization for eight of the ten pathways, including: L‐tryptophan degradation IX (*F*_1,42_ = 10.19, *p* = 0.0027), L‐cysteine biosynthesis VI (*F*_1,42_ = 20.53, *p* < 0.001), assimilatory sulfate reduction I (*F*_1,42_ = 6.66, *p* = 0.0134), allantoin degradation (*F*_1,42_ = 112.05, *p* < 0.001), geranylgeranyl diphosphate biosynthesis II via MEP (*F*_1,42_ = 21.35, *p* < 0.001), fatty acid β‐oxidation II (*F*_1,42_ = 17.41, *p* < 0.001), phytate degradation I (*F*_1,42_ = 12.22, *p* = 0.0011), and putrescine biosynthesis (*F*_1,42_ = 14.58, *p* < 0.001). No significant main effect of sterilization was detected for polyamine biosynthesis II or photorespiration.

The significant effects of species and sterilization interactions were detected for polyamine biosynthesis II (*F*_2,42_ = 10.67, *p* < 0.001), and geranylgeranyl diphosphate biosynthesis II via MEP (*F*_2,42_ = 3.25, *p* = 0.0485). Tukey HSD comparisons showed that the predicted abundance of polyamine biosynthesis II differed between non‐sterilized and sterilized substrates in Fr (*p* = 0.012), but not in Ev or Lc. For geranylgeranyl diphosphate biosynthesis II, a significant NS and S difference was observed in Lc (*p* < 0.001).

For the remaining pathways, significant substrate sterilization differences were detected for L‐tryptophan degradation IX in Lc (*p* = 0.046), L‐cysteine biosynthesis VI (*p* = 0.009), and phytate degradation I (*p* = 0.016) in Fr, and putrescine biosynthesis in Ev (*p* = 0.038). Allantoin degradation showed consistently lower predicted abundance in sterilized substrates across all three plant species (Tukey‐adjusted *p* ≤ 0.001).

Complete metabolic pathways for individual biological replicates are available in Dataset S2.

## 4 Discussion

Rare microbial species have become more recognizable as awareness of their crucial role in ecosystem processes and community assembly across environments has grown (Cena et al. 2021; Chakraborty et al. 2025; Jousset et al. 2017; Pascoal et al. 2021). Here, we investigated extensive green roof substrate microbiome structure based on differently treated growing media, sterilized and non‐sterilized substrate, and the introduction of plant species (*Echium vulagre, Lotus corniculatus*, and *Festuca rubra* ), representing three functional groups: forb, legume, and grass. In the context of the presented results, particular importance is attributed to the reference to plant functional traits summarized in Table 1, which provide a deeper understanding of the mechanisms underlying microbial selection. Variation in life‐history strategies from *Echium vulgare* with specific photosynthetic parameters and pigment contents, through *Lotus corniculatus* dependent on nitrogen‐fixing symbioses, to *Festuca rubra* with distinct biomass allocation and growth dynamics, translated into different patterns of microbiome recruitment. Traits such as photosynthetic efficiency, chlorophyll and anthocyanin content, gas exchange rates, and the ratio of aboveground to belowground biomass indicate diverse strategies for resource management and stress response, most likely directly influencing both the quantity and quality of root exudates and the nature of rhizosphere interactions (Egidi et al. 2025; Singh et al. 2026).

The results support the hypothesis that plant species and the initial microbial state of the substrate are associated with distinct microbial patterns in extensive green roof substrates. The occurrence of plant‐specific discriminant taxa across both fungal and bacterial communities indicates that the three plant species were not associated with an interchangeable microbial assemblage. This finding is consistent with evidence that plant community composition can contribute to the structuring of microbial assemblages in green roofs, although the strength of this relationship may depend on substrate properties and the history of vegetation establishment (Hoch et al. 2019; McGuire et al. 2015; Van Dijck et al. 2024).

### 4.1 Substrate Sterilization

In this study, substrate sterilization was used to create contrasting initial microbial conditions and to assess their contribution to plant‐associated microbiome community structure. Although sterilization of green roof substrates has not previously been used specifically to examine microbial community structure, sterilization of substrates before green roof installation has been reported as a common practice (Fulthorpe et al. 2018). The contrast between sterile and non‐sterile substrates therefore provided an experimental framework for examining plant‐associated community assembly under different initial microbial conditions. This is consistent with the concept of priority effects, whereby the initial microbial inoculum can influence subsequent microbial establishment and community assembly, a mechanism considered particularly relevant to green roof substrates (Hoch et al. 2019; Table S7). Although autoclaving effectively reduces microbial communities in soil and substrate material, it may also alter physicochemical properties and processes related to soil structure and water distribution (Szada‐Borzyszkowska et al. 2024; Vu et al. 2022). Differences in plant responses to sterilized substrates have been reported previously, including increased shoot biomass of *Zea mays* (Rusinowski et al. 2019), whereas responses may depend on plant functional characteristics and microbial symbioses (Heather and Lowther 1992; Boldt‐Burisch and Naeth 2017). Other studies have likewise demonstrated variable plant responses to soil sterilization depending on experimental context (Ding et al. 2023; He et al. 2022; Lorenz et al. 2025; Redouane et al. 2021). Sterilization served primarily as an experimental contrast that allowed plant‐associated microbial patterns to be evaluated against different initial microbial conditions.

### 4.2 Plant Identity, Microbial Differentiation, and Functional Potential

The differences observed among plant treatments indicate that plant identity was associated with distinct patterns of substrate‐associated bacterial and fungal composition. The enrichment of specific taxa under individual plant and substrate variants may reflect differences in the ecological niches associated with particular plant species (Chun et al. 2025), although the present data do not allow causal mechanisms of microbial recruitment or selection to be established. Such plant‐specific patterns are consistent with the view that plant functional traits can contribute to variation in soil microbial communities (Lajoie and Kembel 2019), particularly in environmentally selective systems such as extensive green roofs (Aloisio et al. 2017; Speak et al. 2013). Egidi et al. (2025) similarly emphasized that both soil properties and plant characteristics can shape the diversity, composition, and functions of plant‐associated soil microbial communities. Importantly, these associations varied between sterile and non‐sterile substrates, suggesting that plant‐associated microbial patterns may depend partly on the initial microbial community, consistent with the context‐dependent nature of plant–microbe interactions reported by Song et al. (2025). The co‐occurrence patterns further suggest that plant‐associated differences may extend beyond taxonomic composition to the structure of microbial associations. This is broadly consistent with previous evidence that microbial communities in green roof substrates are influenced by vegetation characteristics (Hoch et al. 2019; Van Dijck et al. 2024), and suggests that substrate sterilization may modify these associations in a plant‐dependent manner.

The results further support previous evidence that plant identity is associated with variation in plant‐associated microbiomes and plant–microbe interactions (Bakker et al. 2020; Berg et al. 2021). Research in natural ecosystems has likewise linked plant identity to nutrient cycling through plant–microorganism interactions (Przybylska et al. 2024), while recent work on plant–soil feedbacks emphasizes that these interactions can vary with environmental conditions (Xi et al. 2025).

In this study, bacterial and fungal communities were analyzed separately, allowing plant‐ and substrate‐associated taxonomic patterns to be evaluated for both major components of the substrate microbiome, consistent with previous green roof microbiome research (Van Dijck et al. 2024). Differences in discriminant microbiota were accompanied by differences in predicted metabolic pathways, suggesting that plant‐associated microbial differentiation was also reflected in variation in predicted functional potential. These pathway‐level patterns should be interpreted as predictions rather than direct measurements of metabolic activity, but they provide a functional context for the observed taxonomic differentiation. Thus, the plant functional trait data presented here go beyond descriptive value by providing a framework for interpreting microbiome assembly and its potential ecological consequences in green roof substrates (Boldt‐Burisch and Naeth 2017).

Because each functional group was represented by a single species, the effects of species identity and functional group are confounded in our experimental design. Therefore, our conclusions are limited to the selected species and should not be interpreted as evidence of plant functional group effects, but rather as effects associated with plant identity. Furthermore, because only substrate microbial communities were analysed, our results do not allow direct inference regarding the reconstruction of root‐associated microbiomes. Future studies integrating soil, rhizosphere, and root microbiomes will be necessary to directly test these mechanisms. Despite these limitations, the observed species‐associated microbial differentiation, together with the corresponding variation in predicted functional potential, provides a basis for further investigation of the mechanisms linking plant identity with microbiome assembly in extensive green roof substrates.

## 5 Conclusions

This study demonstrates that plant identity was associated with distinct bacterial and fungal community patterns in extensive green roof substrates, while these associations depended partly on the initial microbial state of the substrate. Integrating discriminant microbiota with predicted metabolic pathways provided complementary taxonomic and functional perspectives on plant‐associated microbiome differentiation, while the co‐occurrence analysis further highlighted variation in microbial associations. By combining plant functional traits with microbial community analyses, this study highlights the value of integrating plant ecology and microbiology to better understand the biological complexity of extensive green roof systems. These findings provide a basis for considering vegetation and substrate microbiomes jointly in the design and management of more resilient green roof ecosystems.

## Author Contributions

**Katarzyna Larysz:** conceptualization, funding acquisition, investigation, validation, project administration, visualization, formal analysis, writing – original draft, writing – review and editing, resources. **Michał Ludynia:** validation, visualization, methodology, formal analysis, writing – review and editing, resources, supervision. **Artur Trzebny:** investigation, visualization, methodology, writing – review and editing, software. **Wiktoria Klencz:** investigation, writing – review and editing. **Natalia Malcherczyk:** writing – review and editing, resources. **Edyta Sierka:** conceptualization, funding acquisition, validation, project administration, methodology, formal analysis, writing – original draft, writing – review and editing, resources, supervision.

## Funding

The article co‐funded by the project: National Science Centre, Poland (Grant No. 2020/39/B/ST10/00862), and Student Research Grants of the European City of Science Katowice (Grant No. 119118), and “jUŚt transition—Potencjał Uniwersytetu Śląskiego podstawą Sprawiedliwej Transformacji regionu” (FESL.10.25‐IZ.01.0369/23‐003). The project is implemented under the European Funds for Silesia 2021–2027. Program co‐financed by the Just Transition Fund and Director's Reserve of the Institute of Biology, Biotechnology and Environmental Protection of the University of Silesia in Katowice.

## Conflicts of Interest

The authors declare no conflicts of interest.

## Supporting information


**Dataset: S1** comprising SparCC correlation results for microbial co‐occurrence network analysis across six experimental groups.
**Dataset: S2** comprising PICRUSt2‐predicted metabolic pathway abundances across individual biological replicates. The datasets are provided in separate worksheets.

**Table S1:** Properties comparison of green roof substrate before and after the experiment.
**Table S2:** Topological properties of the network of co‐occurrence of substrate microorganisms in different domains.
**Table S3:** Discriminant bacterial genera identified by LEfSe analysis based on V4 taxonomic abundance profiles.
**Table S4:** Discriminant fungal genera identified by LEfSe analysis based on ITS taxonomic abundance profiles.
**Table S5:** Two‐way ANOVA of PICRUSt2‐predicted plant‐relevant microbial pathways in relation to plant species and substrate sterilization.
**Table S6:** Estimated marginal means and Tukey HSD post hoc comparisons of PICRUSt2‐predicted plant‐relevant microbial pathways between non‐sterilized and sterilized substrates.
**Table S7:** Substrate parameters and their relevance to the microbiome.
**Figure S1:** Rarefaction curves based on the Shannon diversity index for bacterial communities (16S rRNA gene sequencing). Each curve represents an individual sample and shows the relationship between sequencing depth and estimated within‐sample (alpha) diversity.
**Figure S2:** Rarefaction curves based on the Shannon diversity index for fungal communities (ITS amplicon sequencing). Each curve represents an individual sample and shows the relationship between sequencing depth and estimated within‐sample (alpha) diversity.
**Figure S3:** Alpha diversity (Shannon index) of bacterial communities across experimental groups. Statistical comparisons were performed using the Kruskal–Wallis test followed by pairwise comparisons with Benjamini–Hochberg correction.
**Figure S4:** Alpha diversity of fungal communities across experimental groups. Statistical comparisons were performed using the Kruskal–Wallis test followed by pairwise comparisons with Benjamini–Hochberg correction.
**Figure S5:** LEfSe identification of discriminant bacterial genera associated with experimental groups.
**Figure S6:** LEfSe identification of discriminant fungal genera associated with experimental groups.

## Data Availability

The raw data with documentation are openly available in NCBI under BioProject accession number PRJNA1479967.

## References

[emi470451-bib-0001] Abarenkov, K. , R. H. Nilsson , K.‐H. Larsson , et al. 2023. “The UNITE Database for Molecular Identification and Taxonomic Communication of Fungi and Other Eukaryotes: Sequences, Taxa and Classifications Reconsidered.” Nucleic Acids Research 51: D495–D504. 10.1093/nar/gkad1039. PMC10767974 37953409

[emi470451-bib-0002] Aloisio, J. M. , M. I. Palmer , M. A. Giampieri , A. R. Tuininga , and J. D. Lewis . 2017. “Spatially Dependent Biotic and Abiotic Factors Drive Survivorship and Physical Structure of Green Roof Vegetation.” Ecological Applications 27: 297–308. 10.1002/eap.1444. 28052509

[emi470451-bib-0003] Ampim, P. A. Y. , J. J. Sloan , R. I. Cabrera , D. A. Harp , and F. H. Jaber . 2010. “Green Roof Growing Substrates: Types, Ingredients, Composition and Properties.” Journal of Environmental Horticulture 28: 244–252.

[emi470451-bib-0004] Bakker, P. A. H. M. , R. L. Berendsen , J. A. Van Pelt , et al. 2020. “The Soil‐Borne Identity and Microbiome‐Assisted Agriculture: Looking Back to the Future.” Molecular Plant 13: 1394–1401. 10.1016/j.molp.2020.09.017. 32979564

[emi470451-bib-0005] Bengtsson‐Palme, J. , M. Ryberg , M. Hartmann , et al. 2013. “Improved Software Detection and Extraction of ITS1 and ITS2 From Ribosomal ITS Sequences of Fungi and Other Eukaryotes for Analysis of Environmental Sequencing Data.” Methods in Ecology and Evolution 4: 914–919.

[emi470451-bib-0006] Berg, G. , P. Kusstatscher , A. Abdelfattah , T. Cernava , and K. Smalla . 2021. “Microbiome Modulation—Toward a Better Understanding of Plant Microbiome Response to Microbial Inoculants.” Frontiers in Microbiology 12: 650610. 10.3389/fmicb.2021.650610. PMC8060476 33897663

[emi470451-bib-0007] Biela, M. , and E. Sierka . 2023. “Using Plants of Novel Ecosystems as Resources to Create Green Roofs in Cities' Adaptation to the Climate Change Process.” Studia Ecologiae et Bioethicae 21: 53–61.

[emi470451-bib-0009] Boldt‐Burisch, K. M. , and M. A. Naeth . 2017. “Mycorrhization Affects Root Distribution of *Lotus corniculatus* in the Rhizosphere.” Frontiers in Rhizosphere 4: 36–47. 10.1016/j.rhisph.2017.06.003.

[emi470451-bib-0010] Bolyen, E. , J. R. Rideout , M. R. Dillon , et al. 2019. “Reproducible, Interactive, Scalable and Extensible Microbiome Data Science Using QIIME 2.” Nature Biotechnology 37: 852–857. 10.1038/s41587-019-0209-9 PMC7015180 31341288

[emi470451-bib-0011] Borowik, A. , J. Wyszkowska , and A. Gałązka . 2019. “Role of *Festuca rubra* and *Festuca arundinacea* in Determining the Functional and Genetic Diversity of Microorganisms and the Enzymatic Activity in the Soil Polluted With Diesel Oil.” Environmental Science and Pollution Research 26: 27738–27751. 10.1007/s11356-019-05888-3. PMC6791909 31338761

[emi470451-bib-0012] Borozan, A. , D. Raba , M. V. Popa , et al. 2020. “Variation of the Bacterial Community in the Rhizosphere of *Lotus corniculatus* L. in the Four Years of Culture.” Journal of Horticulture, Forestry and Biotechnology 23: 22–28.

[emi470451-bib-0013] Callahan, B. J. , P. J. McMurdie , M. J. Rosen , A. W. Han , A. J. A. Johnson , and S. P. Holmes . 2016. “DADA2: High‐Resolution Sample Inference From Illumina Amplicon Data.” Nature Methods 13: 581–583. 10.1038/nmeth.3869 PMC4927377 27214047

[emi470451-bib-0014] Cao, Y. , Q. Dong , D. Wang , P. Zhang , Y. Liu , and C. Niu . 2022. “microbiomeMarker: An R/Bioconductor Package for Microbiome Marker Identification and Visualization.” Bioinformatics 38, no. 16: 4027–4029. 10.1093/bioinformatics/btac438. 35771644

[emi470451-bib-0015] Caporaso, J. G. , C. L. Lauber , W. A. Walters , et al. 2011. “Global Patterns of 16S rRNA Diversity at a Depth of Millions of Sequences Per Sample.” Proceedings of the National Academy of Sciences of the United States of America 108: 4516–4522. 10.1073/pnas.1000080107 PMC3063599 20534432

[emi470451-bib-0016] Cena, J. A. D. , J. Zhang , D. Deng , et al. 2021. “Low‐Abundant Microorganisms: The Human Microbiome's Dark Matter, a Scoping Review.” Frontiers in Cellular and Infection Microbiology 11: 689197. 10.3389/fcimb.2021.689197. PMC8201079 34136418

[emi470451-bib-0017] Cerovic, Z. G. , G. Masdoumier , N. B. Ghozlen , and G. Latouche . 2012. “A New Optical Leaf‐Clip Meter for Simultaneous Non‐Destructive Assessment of Leaf Chlorophyll and Epidermal Flavonoids.” Physiologia Plantarum 146: 251–260. 10.1111/j.1399-3054.2012.01639.x PMC3666089 22568678

[emi470451-bib-0018] Chakraborty, D. , A. Jousset , Z. Wei , and S. Banerjee . 2025. “Rare Taxa in the Core Microbiome.” Trends in Microbiology 33: 727–737. 10.1016/j.tim.2025.03.002. 40155212

[emi470451-bib-0019] Child, H. T. , L. Wierzbicki , G. R. Joslin , et al. 2024. “Comparative Evaluation of Soil DNA Extraction Kits for Long Read Metagenomic Sequencing.” Microbiology Society 6: 000868. 10.1099/acmi.0.000868.v3 PMC11432601 39346682

[emi470451-bib-0020] Chun, S. J. , Y. Cui , and J. Kim . 2025. “Multidimensional Sampling Framework Reveals Plant‐Driven Effects on Microbial Spatial Heterogeneity and Niche Differentiation in a Natural Ecosystem.” Environmental Microbiomes 20: 125. 10.1186/s40793-025-00793-4. PMC12492763 41044628

[emi470451-bib-0021] Corcoz, L. , F. Păcurar , V. Pop‐Moldovan , I. Vaida , V. Stoian , and R. Vidican . 2022. “Mycorrhizal Patterns in the Roots of Dominant *Festuca rubra* in a High‐Natural‐Value Grassland.” Plants 11: 112. 10.3390/plants11010112 PMC8747109 35009115

[emi470451-bib-0022] Csorba, C. , N. Rodić , L. Antonielli , et al. 2024. “Soil pH, Developmental Stages and Geographical Origin Differently Influence the Root Metabolomic Diversity and Root‐Related Microbial Diversity of *Echium vulgare* From Native Habitats.” Frontiers in Plant Science 15: 1369754. 10.3389/fpls.2024.1369754. PMC11232435 38984162

[emi470451-bib-0023] Ding, J. Y. M. , L. S. Ho , J. Ibrahim , C. K. Teh , and K. M. Goh . 2023. “Impact of Sterilization and Chemical Fertilizer on the Microbiota of Oil Palm Seedlings.” Frontiers in Microbiology 14: 1091755. 10.3389/fmicb.2023.1091755. PMC10172575 37180248

[emi470451-bib-0024] Douglas, G. M. , V. J. Maffei , J. R. Zaneveld , et al. 2020. “PICRUSt2 for Prediction of Metagenome Functions.” Nature Biotechnology 38: 685–688. 10.1038/s41587-020-0548-6. PMC7365738 32483366

[emi470451-bib-0025] Edgar, R. C. 2018. “UNCROSS2: Identification of Cross‐Talk in 16S rRNA OTU Tables.”

[emi470451-bib-0026] Egidi, E. , D. Bristol , and K. Hassan . 2025. “The Influence of Plant Traits in Soil Microbial Communities Varies Between Arid and Mesic Grasslands.” Plant and Soil 509: 501–521. 10.1007/s11104-024-06876-4.

[emi470451-bib-0027] Farah, G. , G. Pérez , A. Ballesta , and M. el Bachawati . 2024. “Green Roof Substrates Influencing the Provision of Ecosystem Services: A Review.” Journal of Environmental Chemical Engineering 12: 114795. 10.1016/j.jece.2024.114795.

[emi470451-bib-0030] Fulthorpe, R. , J. S. MacIvor , P. Jia , and S.‐L. E. Yasui . 2018. “The Green Roof Microbiome: Improving Plant Survival for Ecosystem Service Delivery.” Frontiers in Ecology and Evolution 6: 5. 10.3389/fevo.2018.00005.

[emi470451-bib-0031] Garofalo, S. , S. Giovagnoli , M. Orsoni , F. Starita , and M. Benassi . 2022. “Interaction Effect: Are You Doing the Right Thing?” PLoS One 17, no. 7: e0271668. 10.1371/journal.pone.0271668. PMC9299307 35857797

[emi470451-bib-0032] Getter, K. L. , and D. B. Rowe . 2006. “The Role of Extensive Green Roofs in Sustainable Development.” HortScience 41: 1276–1285.

[emi470451-bib-0033] Glöckner, F. O. , P. Yilmaz , C. Quast , et al. 2017. “25 Years of Serving the Community With Ribosomal RNA Gene Reference Databases and Tools.” Journal of Biotechnology 261: 169–176. 10.1016/j.jbiotec.2017.06.1198 28648396

[emi470451-bib-0034] Goltsev, V. , H. M. Kalaji , W. Bąba , C. Lu , and I. G. Greberkin . 2012. “Drought‐Induced Modifications of Photosynthetic Electron Transport in Intact Leaves: Analysis and Use of Neural Networks as a Tool for a Rapid Non‐Invasive Estimation.” Biochimica Et Biophysica Acta (BBA) ‐ Bioenergetics 1817: 1490–1498. 10.1016/j.bbabio.2012.04.018. 22609146

[emi470451-bib-0036] Hannon, G. J. 2010. “FASTX‐Toolkit.” http://hannonlab.cshl.edu.

[emi470451-bib-0037] He, H. , L. Yu , X. Yang , et al. 2022. “Effects of Different Soils on the Biomass and Photosynthesis of *Rumex nepalensis* in Subalpine Region of Southwestern China.” Forests 13: 73. 10.3390/f13010073.

[emi470451-bib-0038] Heather, P. N. , and W. L. Lowther . 1992. “Response of *Lotus corniculatus* Nodulation and Growth to Heavy Metal Stress.” New Zealand Journal of Agricultural Research 35: 2050. 10.1080/03081945.1992.10419248.

[emi470451-bib-0039] Hector, A. , S. Von Felten , and B. Schmid . 2010. “Analysis of Variance With Unbalanced Data: An Update for Ecology & Evolution.” Journal of Animal Ecology 79: 308–316. 10.1111/j.1365-2656.2009.01634.x. 20002862

[emi470451-bib-0040] Heim, A. , and J. Lundholm . 2022. “Changes in Plant Community Composition and Functional Plant Traits Over a Four‐Year Period on an Extensive Green Roof.” Journal of Environmental Management 304: 114154. 10.1016/j.jenvman.2021.114154. 34864420

[emi470451-bib-0042] Hoch, J. M. K. , M. E. Rhodes , K. L. Shek , et al. 2019. “Soil Microbial Assemblages Are Linked to Plant Community Composition and Contribute to Ecosystem Services on Urban Green Roofs.” Frontiers in Ecology and Evolution 7: 198. 10.3389/fevo.2019.00198.

[emi470451-bib-0043] Jousset, A. , C. Bienhold , A. Chatzinotas , et al. 2017. “Where Less May Be More: How the Rare Biosphere Pulls Ecosystems Strings.” ISME Journal 11: 853–862. 10.1038/ismej.2016.174. PMC5364357 28072420

[emi470451-bib-0044] Kader, S. , S. Chadalavada , L. Jaufer , V. Spalevic , and B. Dudic . 2022. “Green Roof Substrates—A Literature Review.” Frontiers in Built Environment 8: 1019362. 10.3389/fbuil.2022.1019362.

[emi470451-bib-0045] Kalaji, H. M. , G. Schansker , R. J. Ladle , et al. 2014. “Frequently Asked Questions About In Vivo Chlorophyll Fluorescence: Practical Issues.” Photosynthesis Research 122: 121–158. 10.1007/s11120-014-0024-6. PMC4210649 25119687

[emi470451-bib-0046] Kanehisa, M. 2019. “Toward Understanding the Origin and Evolution of Cellular Organisms.” Protein Science 28: 1947–1951. 10.1002/pro.3715. PMC6798127 31441146

[emi470451-bib-0047] Kanehisa, M. , M. Furumichi , Y. Sato , M. Ishiguro‐Watanabe , and M. Tanabe . 2021. “KEGG: Integrating Viruses and Cellular Organisms.” Nucleic Acids Research 49: D545–D551. 10.1093/nar/gkaa970. PMC7779016 33125081

[emi470451-bib-0048] Kargas, G. , N. Ntoulas , and A. Tsapatsouli . 2023. “Use of a Dielectric Sensor for Salinity Determination on an Extensive Green Roof Substrate.” Sensors 23: 5802. 10.3390/s23135802. PMC10346240 37447651

[emi470451-bib-0049] Klemow, K. M. , D. R. Clements , P. F. Threadgill , and P. B. Cavers . 2002. “The Biology of Canadian Weeds. 116. *Echium vulgare* L.” Canadian Journal of Plant Science 82: 235–248. 10.4141/P01-058.

[emi470451-bib-0051] Kosová, V. , T. Hájek , V. Hadincová , and Z. Münzbergová . 2022. “The Importance of Ecophysiological Traits in Response of *Festuca rubra* to Changing Climate.” Physiologia Plantarum 174: e13608. 10.1111/ppl.13608. 34837234

[emi470451-bib-0052] Lajoie, G. , and S. W. Kembel . 2019. “Making the Most of Trait‐Based Approaches for Microbial Ecology.” Trends in Microbiology 27: 814–823. 10.1016/j.tim.2019.06.003 31296406

[emi470451-bib-0053] Lane, D. J. , B. Pace , G. J. Olsen , D. A. Stahl , M. L. Sogin , and N. R. Pace . 1985. “Rapid Determination of 16S Ribosomal RNA Sequences for Phylogenetic Analyses.” Proceedings of the National Academy of Sciences of the United States of America 82: 6955–6959. 10.1073/pnas.82.20.6955 PMC391288 2413450

[emi470451-bib-0054] Langille, M. G. I. , J. Zaneveld , J. G. Caporaso , et al. 2013. “Predictive Functional Profiling of Microbial Communities Using 16S rRNA Marker Gene Sequences.” Nature Biotechnology 31: 814–821. 10.1038/nbt.2676. PMC3819121 23975157

[emi470451-bib-0102] Larysz, K. , and E. Sierka . 2024. “Mikrobiom Dachów Zielonych: Stan Wiedzy i Przyszłe Wyzwania.” In Mikrobiologia w praktyce – odkrycia i innowacje w badaniach środowiskowych i medycznych, 1, 7–19. Tygiel Academic Publishing House. https://bc.wydawnictwo‐tygiel.pl/publikacja/9B415EC2‐037F‐F159‐99A9‐1B5601769286.

[emi470451-bib-0055] Latini, A. , I. Papagni , L. Gatti , et al. 2022. “ *Echium vulgare* and *Echium plantagineum* : A Comparative Study to Evaluate Their Inclusion in Mediterranean Urban Green Roofs.” Sustainability 14: 9581. 10.3390/su14159581.

[emi470451-bib-0056] Lavorel, S. , and E. Garnier . 2002. “Predicting Changes in Community Composition and Ecosystem Functioning From Plant Traits: Revisiting the Holy Grail.” Functional Ecology 16: 545–556. 10.1046/j.1365-2435.2002.00664.x.

[emi470451-bib-0058] Lorenz, C. , E. Vitale , C. Piraino , S. Castaldi , R. Isticato , and C. Arena . 2025. “Enhancing Plant Productivity by Nature‐Based Solutions: Exploring Plant Growth Promoting Bacteria (PGPB) Application in Sterilized and Non‐Sterilized Technosol for Urban Farming.” Scientia Horticulturae 349: 114262. 10.1016/j.scienta.2025.114262.

[emi470451-bib-0059] Lundholm, J. T. 2006. “Green Roofs and Facades: A Habitat Template Approach.” Urban Habitats 4: 87–101.

[emi470451-bib-0060] McGuire, K. L. , S. Payne , G. Orazi , and M. I. Palmer . 2015. “Bacteria and Fungi in Green Roof Ecosystems.” In Green Roof Ecosystems, edited by R. Sutton , 175–191. Springer. 10.1007/978-3-319-14983-7_7.

[emi470451-bib-0063] Ndayambaje, P. , J. S. MacIvor , and M. W. Cadotte . 2024. “Plant Diversity on Green Roofs: A Review of the Ecological Benefits, Challenges, and Best Management Practices.” Nature‐Based Solutions 6: 100162. 10.1016/j.nbsj.2024.100162.

[emi470451-bib-0064] Oberndorfer, E. , J. Lundholm , B. Bass , et al. 2007. “Green Roofs as Urban Ecosystems: Ecological Structures, Functions, and Services.” Bioscience 57: 823–833. 10.1641/B571005.

[emi470451-bib-0065] Pascoal, F. , R. Costa , and C. Magalhães . 2021. “The Microbial Rare Biosphere: Current Concepts, Methods and Ecological Principles.” FEMS Microbiology Ecology 97: fiaa227. 10.1093/femsec/fiaa227. 33175111

[emi470451-bib-0066] Petrova, S. , I. Velcheva , B. Nikolov , et al. 2022. “Nature‐Based Solutions for the Sustainable Management of Urban Soils and Quality of Life Improvements.” Land 11: 569. 10.3390/land11040569.

[emi470451-bib-0067] Przybylska, M. S. , C. Violle , D. Vile , et al. 2024. “Can Plants Build Their Niche Through Modulation of Soil Microbial Activities Linked With Nitrogen Cycling? A Test With *Arabidopsis thaliana* .” New Phytologist 243: 620–635. 10.1111/nph.19870. 38812269

[emi470451-bib-0068] Quast, C. , E. Pruesse , P. Yilmaz , et al. 2013. “The SILVA Ribosomal RNA Gene Database Project: Improved Data Processing and Web‐Based Tools.” Nucleic Acids Research 41: D590–D596. 10.1093/nar/gks1219 PMC3531112 23193283

[emi470451-bib-0069] R Core Team . 2022. R: A Language and Environment for Statistical Computing. R Foundation for Statistical Computing. https://www.R‐project.org.

[emi470451-bib-0070] Redouane, E. M. , M. Lahrouni , J. C. Martins , et al. 2021. “Protective Role of Native Rhizospheric Soil Microbiota Against the Exposure to Microcystins Introduced Into Soil–Plant System via Contaminated Irrigation Water and Health Risk Assessment.” Toxins (Basel) 13: 118. 10.3390/toxins13020118. PMC7914557 33562776

[emi470451-bib-0071] Revelle, W. 2025. Psych: Procedures for Psychological, Psychometric, and Personality Research. R Package Version 2.5.6. Northwestern University. https://CRAN.R‐project.org/package=psych.

[emi470451-bib-0072] Rusinowski, S. , A. Szada‐Borzyszkowska , P. Zieleźnik‐Rusinowska , et al. 2019. “How Autochthonous Microorganisms Influence Physiological Status of *Zea mays* L. Cultivated on Heavy Metal Contaminated Soils?” Environmental Science and Pollution Research 26: 4746–4763. 10.1007/s11356-018-3923-9. PMC6394448 30565117

[emi470451-bib-0073] Seaborg, D. 2022. “Plants Are Ecosystem Engineers That Aid Other Life and Are Linked to It.” In How Life Increases Biodiversity, edited by F. Ouimane , 1st ed., 179–194. CRC Press. 10.1201/9780429440137-7.

[emi470451-bib-0074] Segata, N. , J. Izard , L. Waldron , et al. 2011. “Metagenomic Biomarker Discovery and Explanation.” Genome Biology 12, no. 6: R60. 10.1186/gb-2011-12-6-r60. PMC3218848 21702898

[emi470451-bib-0076] Singh, P. K. , C. S. C. Martins , R. H. Jayaramaiah , et al. 2026. “Leaf Microbiome Assembly Is Linked to Plant Phylogeny.” Plant and Soil 520: 1177–1192. 10.1007/s11104-026-08318-9.

[emi470451-bib-0077] Song, S. , X. Yang , J. Zhu , R. Tang , and Z. Tang . 2025. “Plant Functional Diversity Regulates the Composition and Diversity of Soil Microbial Communities in Temperate Grasslands of Northern China.” Functional Ecology 39: 2314–2326. 10.1111/1365-2435.70103.

[emi470451-bib-0078] Speak, A. F. , J. J. Rothwell , S. J. Lindley , and C. L. Smith . 2013. “Reduction of the Urban Cooling Effects of an Intensive Green Roof due to Vegetation Damage.” Urban Climate 3: 40–55. 10.1016/j.uclim.2013.01.001.

[emi470451-bib-0080] Steenaerts, L. , F. Rineau , T. Artois , et al. 2026. “The Influence of Green Roof Features on Their Carbon and Nutrient Stocks.” Urban Ecosystems 29: 32. 10.1007/s11252-025-01898-x.

[emi470451-bib-0081] Stirbet, A. , D. Lazár , and J. Kromdijk . 2018. “Chlorophyll a Fluorescence Induction: Can Just a One‐Second Measurement Be Used to Quantify Abiotic Stress Responses?” Photosynthetica 56: 86–104.

[emi470451-bib-0082] Sujkowska‐Rybkowska, M. , D. Kasowska , K. Gediga , J. Banasiewicz , and T. Stępkowski . 2020. “ * Lotus corniculatus‐*Rhizobia Symbiosis Under ni, co and Cr Stress on Ultramafic Soil.” Plant and Soil 451: 459–484. 10.1007/s11104-020-04546-9.

[emi470451-bib-0083] Sun, C. , W. Zhang , G. Zhao , et al. 2024. “Mapping Rapeseed ( *Brassica napus* L.) Aboveground Biomass in Different Periods Using Optical and Phenotypic Metrics Derived From UAV Hyperspectral and RGB Imagery.” Frontiers in Plant Science 15: 1504119. 10.3389/fpls.2024.1504119. PMC11659009 39711583

[emi470451-bib-0084] Szada‐Borzyszkowska, A. , J. Krzyżak , S. Rusinowski , F. Magurno , and M. Pogrzeba . 2024. “Inoculation With Arbuscular Mycorrhizal Fungi Supports the Uptake of Macronutrients and Promotes the Growth of *Festuca ovina* L. and *Trifolium medium* L., a Candidate Species for Green Urban Infrastructure.” Plants (Basel) 13: 2620. 10.3390/plants13182620. PMC11434852 39339595

[emi470451-bib-0085] Todeschini, C. C. , and A. G. Fett‐Neto . 2025. “Life at the Top: Extensive Green Roof Plant Species and Their Traits for Urban Use.” Plants (Basel) 14: 735. 10.3390/plants14050735. PMC11902263 40094684

[emi470451-bib-0086] Toju, H. , A. S. Tanabe , S. Yamamoto , and H. Sato . 2012. “High‐Coverage ITS Primers for the DNA‐Based Identification of Ascomycetes and Basidiomycetes in Environmental Samples.” PLoS One 7: e40863. 10.1371/journal.pone.0040863 PMC3395698 22808280

[emi470451-bib-0087] Trivedi, P. , J. E. Leach , S. G. Tringe , T. Sa , and B. K. Singh . 2020. “Plant–Microbiome Interactions: From Community Assembly to Plant Health.” Nature Reviews Microbiology 18: 607–621. 10.1038/s41579-020-0412-1. 32788714

[emi470451-bib-0088] Tsimilli‐Michael, M. , and R. J. Strasser . 2004. “Photosystem II Adaptation to Changing Light Conditions as a Process Leading to Optimality/Stability: A Theoretical Approach and a Model Using Different Types of Conformational/State Changes.” Acta Physiologiae Plantarum 26: 176–177.

[emi470451-bib-0089] van der Heijden, M. , J. Klironomos , and M. Ursic . 1998. “Mycorrhizal Fungal Diversity Determines Plant Biodiversity, Ecosystem Variability and Productivity.” Nature 396: 69–72. 10.1038/23932.

[emi470451-bib-0090] Van Dijck, T. , V. Stevens , L. Steenaerts , et al. 2024. “Green Roof Substrate Microbes Compose a Core Community of Stress‐Tolerant Taxa.” Microorganisms 12: 1261. 10.3390/microorganisms12071261. PMC11279297 39065029

[emi470451-bib-0091] Vannucchi, F. , C. Bibbiani , C. Caudai , and F. Bretzel . 2023. “Mediterranean Extensive Green Roof Self‐Sustainability Mediated by Substrate Composition and Plant Strategy.” Horticulturae 9: 1117. 10.3390/horticulturae9101117.

[emi470451-bib-0092] Vu, E. , G. E. Schaumann , and C. Buchmann . 2022. “The Contribution of Microbial Activity to Soil–Water Interactions and Soil Microstructural Stability of a Silty Loam Soil Under Moisture Dynamics.” Geoderma 417: 115822. 10.1016/j.geoderma.2022.115822.

[emi470451-bib-0093] Wala, M. , J. Kołodziejek , and J. Patykowski . 2021. “Nitrogen Signals and Their Ecological Significance for Seed Germination of Ten Psammophilous Plant Species From European Dry Acidic Grasslands.” PLoS One 16: e0244737. 10.1371/journal.pone.0244737. PMC7781371 33395438

[emi470451-bib-0095] Wolf, D. C. , and H. D. Skipper . 1994. “Soil Sterilization.” In Methods of Soil Analysis, edited by R. W. Weaver , 2nd ed., 517–525. Soil Science Society of America. 10.2136/sssabookser5.2.c3.

[emi470451-bib-0096] Xi, N. , J. R. De Long , J. Davison , et al. 2025. “Plant–Soil Microbial Interactions as Modulators of Species Coexistence and Productivity.” Trends in Ecology & Evolution 40: 673–686. 10.1016/j.tree.2025.04.013. 40389331

[emi470451-bib-0098] Yi, Y. , Y. Fang , K. Wu , Y. Liu , and W. Zhang . 2020. “Comprehensive Gene and Pathway Analysis of Cervical Cancer Progression.” Oncology Letters 19: 3316–3332. 10.3892/ol.2020.11439. PMC7074609 32256826

[emi470451-bib-0099] Yilmaz, P. , L. W. Parfrey , P. Yarza , et al. 2014. “The SILVA and ‘All‐Species Living Tree Project (LTP)’ Taxonomic Frameworks.” Nucleic Acids Research 42: 643–648. 10.1093/nar/gkt1209 PMC3965112 24293649

