# Supplementary material for: Plant Identity Shapes Core Microbiomes on Green Roofs
Source: Environ Microbiol Rep. 2026 Oct 30;18(6):e70451. doi: 10.1111/1758-2229.70451 (PMC13720972; doi:10.1111/1758-2229.70451)
Supplement: Supplementary file 1 [file EMI4-18-e70451-s001.docx]

**Environmental Microbiology Reports**

**RESEARCH ARTICLE**

Plant Identity Shapes Core Microbiomes on Green Roofs

**Katarzyna Larysz^1^* | Michał Ludynia^2^ | Artur Trzebny^3^ | Wiktoria Klencz^2^ | Natalia Malcherczyk^2^* | Edyta Sierka^2^**

^1^University of Silesia in Katowice, International Environmental Doctoral School in Sosnowiec, Poland

^2^University of Silesia in Katowice, Faculty of Natural Sciences, Institute of Biology, Biotechnology and Environmental Protection, Poland

^3^Adam Mickiewicz University Poznan, Faculty of Biology, Institute of Molecular Biology and Biotechnology, Molecular Biology Techniques Laboratory, Poland

***Corresponding Authors:** Katarzyna Larysz, katarzyna.larysz@us.edu.pl

Edyta Sierka, edyta.sierka@us.edu.pl

**Supplementary Material
TABLE S1** Properties comparison of green roof substrate before and after the experiment

| **Properties** | **Manufacturer data sheet** | **Bare substrate analyses** |
| --- | --- | --- |
| Material (main components) | expanded shale, expanded clay, lava, pumice, crushed brick, Porlith, and green waste compost (with a variable composition depending on the region) | - |
| Total pore volume | > 60–70 Vol% | - |
| Max. water capacity | ⩾ 35 Vol% | - |
| Salt content | ⩽ 3.5 g·dm^-3^ | 0.26 g·dm^-3^ |
| Organic substances | < 65 g·dm^-3^ | - |
| pH value | 6.0–8.5 | 7.3 |
| Weight when saturated | 1,140–1,440 kg·m^-3^ | - |
| Water permeability | ⩾ 0.6 mm·min^-1^ | - |
| Compaction factor | 1.2 | - |
| Macronutrients  Nitrate nitrogen  Phosphorus  Potassium  Calcium  Magnesium  Accessible sodium  Chloride | - | 14 mg·dm^-3^  192 mg·dm^-3^  120 mg·dm^-3^  1263 mg·dm^-3^  220 mg·dm^-3^  51 mg·dm^-3^  28 mg·dm^-3^ |
| Micronutrients  Copper  Iron  Manganese  Zinc | - | 2.1 mg·dm^-3^  79.6 mg·dm^-3^  18.5 mg·dm^-3^  6.7 mg·dm^-3^ |

**TABLE S2** Topological properties of the network of co-occurrence of substrate microorganisms in different domains

| **Network features** | | **NS-Ev** | **NS-Lc** | **NS-Fr** | **S-Ev** | **S-Lc** | **S-Fr** |
| --- | --- | --- | --- | --- | --- | --- | --- |
| Nodes | Total | 448 | 455 | 509 | 376 | 353 | 362 |
|  | Bacteria | 363 | 370 | 422 | 313 | 299 | 306 |
|  | Fungi | 85 | 85 | 87 | 63 | 54 | 56 |
| Edges | Total | 13715 | 10465 | 12713 | 7024 | 6125 | 10595 |
|  | Bacteria-Bacteria | 12854 | 9085 | 11 569 | 6362 | 5147 | 9680 |
|  | Fungi-Fungi | 339 | 305 | 191 | 169 | 203 | 241 |
|  | Bacteria-Fungi | 522 | 1075 | 953 | 493 | 775 | 674 |
|  | % Bacteria-Bacteria | 93.72 | 86.81 | 91.00 | 90.58 | 84.03 | 91,36 |
|  | % Fungi-Fungi | 2.47 | 2.91 | 1.50 | 2.41 | 3.31 | 2,27 |
|  | % Bacteria-Fungi | 3.81 | 10.27 | 7.50 | 7.02 | 12.65 | 6,36 |
| Positive correlation direction | | 10803 | 6182 | 6 712 | 4183 | 3541 | 5415 |
| Negative correlation direction | | 2912 | 4283 | 6 001 | 2841 | 2584 | 5180 |
| Average degree | | 61,228 | 46 | 49.953 | 37.362 | 34.703 | 58.536 |
| Graph density | | 0,137 | 0.101 | 0.098 | 0.1 | 0.099 | 0.162 |
| Modularity | | 0,396 | 0.393 | 0.431 | 0.432 | 0.466 | 0.38 |

**TABLE S3** Discriminant bacterial genera identified by LEfSe analysis based on V4 taxonomic abundance profiles

| **Genus** | **Enriched group** | **Relative abundance (%)** | **KW p-value** | **Wilcoxon p-value** | **p-adjusted KW** | **LDA score** |
| --- | --- | --- | --- | --- | --- | --- |
| ***Sphingomonas*** | NS-Lc | 10.38106 | 0.00212 | 0.02595 | 0.00212 | 4.89073 |
| ***Brevundimonas*** | S-Ev | 8.71345 | 0.04351 | 0.02994 | 0.04351 | 4.65934 |
| ***Tumebacillus*** | S-Fr | 2.78381 | 0.00003 | 0.00795 | 0.00003 | 4.32992 |
| ***Arenimonas*** | NS-Lc | 2.04052 | 0.00426 | 0.04456 | 0.00426 | 4.24186 |
| ***Paenibacillus*** | S-Lc | 1.47220 | 0.02717 | 0.07121 | 0.02717 | 4.19433 |
| ***Brevibacillus*** | S-Lc | 2.36833 | 0.00021 | 0.01009 | 0.00021 | 4.15902 |
| ***Acidibacter*** | NS-Lc | 1.50693 | 0.00589 | 0.03801 | 0.00589 | 4.12046 |
| *Roseisolibacter* | S-Ev | 1.50225 | 0.02837 | 0.01582 | 0.02837 | 3.99430 |
| *Caulobacter* | S-Ev | 1.42348 | 0.00664 | 0.01958 | 0.00664 | 3.94118 |
| *Chryseolinea* | NS-Fr | 0.91478 | 0.03675 | 0.06574 | 0.03675 | 3.92395 |
| *Cohnella* | S-Lc | 0.87148 | 0.00003 | 0.00437 | 0.00003 | 3.90314 |
| *Pseudoxanthomonas* | NS-Fr | 0.95787 | 0.02871 | 0.04167 | 0.02871 | 3.86664 |
| *Haliangium* | NS-Lc | 0.65678 | 0.03641 | 0.07900 | 0.03641 | 3.74437 |
| *Aquicella* | NS-Fr | 0.54837 | 0.00819 | 0.03652 | 0.00819 | 3.71988 |
| *Pirellula* | NS-Fr | 0.60519 | 0.03240 | 0.03180 | 0.03240 | 3.71182 |
| *Hydrogenophaga* | NS-Fr | 0.44609 | 0.02373 | 0.06574 | 0.02373 | 3.64743 |
| *Ohtaekwangia* | NS-Lc | 0.47295 | 0.01554 | 0.03603 | 0.01554 | 3.60863 |
| *Bryobacter* | NS-Lc | 0.49300 | 0.00448 | 0.03391 | 0.00448 | 3.60732 |
| *Hirschia* | NS-Lc | 0.40606 | 0.01525 | 0.04987 | 0.01525 | 3.53185 |
| *Gaiella* | NS-Lc | 0.30840 | 0.00261 | 0.02160 | 0.00261 | 3.48771 |
| *Nitrospira* | NS-Lc | 0.34112 | 0.03640 | 0.07696 | 0.03640 | 3.43128 |
| *Ahniella* | NS-Lc | 0.30392 | 0.00073 | 0.00982 | 0.00073 | 3.42619 |
| *Salipaludibacillus* | S-Lc | 0.33135 | 0.00149 | 0.05803 | 0.00149 | 3.40648 |
| *Bauldia* | NS-Lc | 0.25158 | 0.01435 | 0.03391 | 0.01435 | 3.34298 |
| *Blastocatella* | NS-Fr | 0.25936 | 0.00281 | 0.04027 | 0.00281 | 3.26866 |
| *Iamia* | NS-Lc | 0.18887 | 0.00574 | 0.01789 | 0.00574 | 3.23523 |
| *Chthoniobacter* | NS-Fr | 0.26992 | 0.04696 | 0.02353 | 0.04696 | 3.22493 |
| *Sphingoaurantiacus* | S-Lc | 0.22099 | 0.00401 | 0.06348 | 0.00401 | 3.20590 |
| *Sphingorhabdus* | NS-Fr | 0.16099 | 0.00934 | 0.07767 | 0.00934 | 3.18770 |
| *Noviherbaspirillum* | NS-Fr | 0.12407 | 0.00212 | 0.02513 | 0.00212 | 3.17090 |
| *Ammoniphilus* | S-Lc | 0.17184 | 0.00607 | 0.03946 | 0.00607 | 3.16693 |
| *Polaromonas* | NS-Fr | 0.11905 | 0.02218 | 0.00259 | 0.02218 | 3.15007 |
| *Rhodanobacter* | NS-Fr | 0.13762 | 0.01318 | 0.04312 | 0.01318 | 3.12202 |
| *Fimbriiglobus* | NS-Fr | 0.12389 | 0.02233 | 0.01379 | 0.02233 | 3.12087 |
| *Sandaracinus* | S-Ev | 0.23912 | 0.03137 | 0.00425 | 0.03137 | 3.11266 |
| *Candidatus_Berkiella* | NS-Fr | 0.10979 | 0.01333 | 0.06278 | 0.01333 | 3.04755 |
| *Pseudorhodoplanes* | S-Ev | 0.12185 | 0.03844 | 0.30112 | 0.03844 | 3.00470 |
| *Phaeodactylibacter* | NS-Lc | 0.11123 | 0.00087 | 0.00949 | 0.00087 | 2.98901 |
| *Dokdonella* | NS-Fr | 0.08983 | 0.00697 | 0.01654 | 0.00697 | 2.95425 |
| *Tepidimicrobium* | S-Lc | 0.10415 | 0.01006 | 0.01582 | 0.01006 | 2.94744 |
| *Dinghuibacter* | NS-Fr | 0.09324 | 0.00756 | 0.00276 | 0.00756 | 2.90773 |
| *Limnobacter* | NS-Fr | 0.08921 | 0.00738 | 0.00080 | 0.00738 | 2.90646 |
| *Aridibacter* | NS-Lc | 0.06432 | 0.04923 | 0.07174 | 0.04923 | 2.78799 |
| *Rubellimicrobium* | NS-Lc | 0.04663 | 0.02972 | 0.09473 | 0.02972 | 2.76594 |
| *Hansschlegelia* | NS-Ev | 0.04942 | 0.00930 | 0.07767 | 0.00930 | 2.74536 |
| *Nannocystis* | NS-Lc | 0.05868 | 0.02094 | 0.16442 | 0.02094 | 2.74528 |
| *Cupriavidus* | S-Lc | 0.06723 | 0.02688 | 0.03056 | 0.02688 | 2.72681 |
| *Zavarzinella* | NS-Fr | 0.05092 | 0.00285 | 0.00625 | 0.00285 | 2.69966 |
| *Marmoricola* | NS-Ev | 0.08559 | 0.02557 | 0.01112 | 0.02557 | 2.69682 |
| *Micromonospora* | NS-Ev | 0.05321 | 0.03611 | 0.51258 | 0.03611 | 2.69266 |
| *Halocella* | S-Lc | 0.07182 | 0.00020 | 0.00052 | 0.00020 | 2.69204 |
| *Skermanella* | NS-Ev | 0.05033 | 0.04235 | 0.10174 | 0.04235 | 2.66421 |
| *Agromyces* | NS-Ev | 0.03463 | 0.03549 | 0.22994 | 0.03549 | 2.59902 |
| *Caldalkalibacillus* | S-Ev | 0.05437 | 0.03086 | 0.02744 | 0.03086 | 2.58820 |
| *Ochrobactrum* | S-Ev | 0.04814 | 0.03779 | 0.00598 | 0.03779 | 2.52971 |
| *Ilumatobacter* | NS-Lc | 0.03141 | 0.00861 | 0.03646 | 0.00861 | 2.52344 |
| *Variovorax* | NS-Lc | 0.04579 | 0.00725 | 0.00208 | 0.00725 | 2.48153 |
| *Hymenobacter* | S-Ev | 0.04219 | 0.03978 | 0.98202 | 0.03978 | 2.45466 |
| *Roseococcus* | S-Ev | 0.02088 | 0.02955 | 0.00351 | 0.02955 | 2.29359 |
| *Phaselicystis* | S-Fr | 0.03615 | 0.01967 | 0.01230 | 0.01967 | 2.29184 |
| *Sulfurifustis* | NS-Lc | 0.02429 | 0.02819 | 0.00202 | 0.02819 | 2.27871 |
| *Rhodopseudomonas* | S-Fr | 0.02165 | 0.03984 | 0.00616 | 0.03984 | 2.21067 |
| *Verrucomicrobium* | NS-Fr | 0.01149 | 0.03295 | 0.09992 | 0.03295 | 2.17653 |
| *Actinotalea* | NS-Lc | 0.00662 | 0.00793 | 0.00009 | 0.00793 | 2.06191 |
| *Tahibacter* | NS-Lc | 0.00678 | 0.00880 | 0.00069 | 0.00880 | 2.05252 |

**TABLE S4** Discriminant fungal genera identified by LEfSe analysis based on ITS taxonomic abundance profiles

| **Genus** | **Enriched group** | **Relative abundance (%)** | **KW p-value** | **Wilcoxon p-value** | **p-adjusted KW** | **LDA score** |
| --- | --- | --- | --- | --- | --- | --- |
| ***Mortierella*** | NS-Ev | 11.41044 | 0.01287 | 0.00544 | 0.01287 | 4.80772 |
| ***Schizothecium*** | NS-Lc | 6.87539 | 0.00002 | 0.05257 | 0.00002 | 4.79858 |
| ***Serendipita*** | NS-Fr | 6.05902 | 0.03053 | 0.07607 | 0.03053 | 4.79705 |
| ***Acremonium*** | S-Fr | 13.16463 | 0.00366 | 0.00543 | 0.00366 | 4.78625 |
| *Ascobolus* | NS-Ev | 5.76196 | 0.00041 | 0.00889 | 0.00041 | 4.56299 |
| *Arthrobotrys* | NS-Lc | 3.64174 | 0.00064 | 0.71682 | 0.00064 | 4.51102 |
| *Arachniotus* | NS-Ev | 3.80093 | 0.00460 | 0.06682 | 0.00460 | 4.47838 |
| *Neopyrenochaeta* | NS-Lc | 2.07776 | 0.01536 | 0.14934 | 0.01536 | 4.34659 |
| *Petriella* | NS-Fr | 0.56191 | 0.04718 | 0.07431 | 0.04718 | 3.85185 |
| *Mycothermus* | NS-Lc | 0.52185 | 0.00086 | 0.00616 | 0.00086 | 3.67018 |
| *Basidiobolus* | NS-Lc | 0.38007 | 0.04513 | 0.60350 | 0.04513 | 3.61693 |
| *Exophiala* | NS-Ev | 0.55438 | 0.03299 | 0.00100 | 0.03299 | 3.45551 |
| *Mycofalcella* | NS-Lc | 0.21250 | 0.03680 | 0.00183 | 0.03680 | 3.30803 |

**TABLE S5** Two-way ANOVA of PICRUSt2-predicted plant-relevant microbial pathways in relation to plant species and substrate sterilization

| **Pathway** | **Effect** | **df** | **F** | **p value** |
| --- | --- | --- | --- | --- |
| **superpathway of polyamine biosynthesis II** | Species | 2 | 7.2422 | 0.00198 |
|  | Sterilization | 1 | 0.15064 | 0.69989 |
|  | Species x Sterilization | 2 | 10.667 | 0.00018 |
| **L-tryptophan degradation IX** | Species | 2 | 3.0559 | 0.05767 |
|  | Sterilization | 1 | 10.192 | 0.00267 |
|  | Species x Sterilization | 2 | 3.1248 | 0.05431 |
| **L-cysteine biosynthesis VI (from L-methionine)** | Species | 2 | 1.2278 | 0.30324 |
|  | Sterilization | 1 | 20.533 | 0.00005 |
|  | Species x Sterilization | 2 | 0.97592 | 0.38523 |
| **assimilatory sulfate reduction I** | Species | 2 | 0.2431 | 0.78529 |
|  | Sterilization | 1 | 6.6612 | 0.01344 |
|  | Species x Sterilization | 2 | 0.18644 | 0.83059 |
| **superpathway of allantoin degradation in plants** | Species | 2 | 0.27749 | 0.75906 |
|  | Sterilization | 1 | 112.05 | p<0.001 |
|  | Species x Sterilization | 2 | 0.39156 | 0.67844 |
| photorespiration | Species | 2 | 1.1664 | 0.32138 |
|  | Sterilization | 1 | 1.8774 | 0.17791 |
|  | Species x Sterilization | 2 | 0.44363 | 0.64468 |
| **superpathway of geranylgeranyl diphosphate biosynthesis II (via MEP)** | Species | 2 | 0.79465 | 0.45841 |
|  | Sterilization | 1 | 21.349 | 0.00004 |
|  | Species x Sterilization | 2 | 3.2544 | 0.04853 |
| **fatty acid &beta;-oxidation II (plant peroxisome)** | Species | 2 | 0.11583 | 0.89091 |
|  | Sterilization | 1 | 17.408 | 0.00015 |
|  | Species x Sterilization | 2 | 0.10734 | 0.89846 |
| **phytate degradation I** | Species | 2 | 0.00174 | 0.99826 |
|  | Sterilization | 1 | 12.224 | 0.00113 |
|  | Species x Sterilization | 2 | 2.4667 | 0.09708 |
| **superpathway of putrescine biosynthesis** | Species | 2 | 1.0336 | 0.36461 |
|  | Sterilization | 1 | 14.581 | 0.00044 |
|  | Species x Sterilization | 2 | 1.3169 | 0.27879 |

**TABLE S6** Estimated marginal means and Tukey HSD post hoc comparisons of PICRUSt2-predicted plant-relevant microbial pathways between non-sterilized and sterilized substrates

| **Pathway** | **Species** | **NS EMM** | **S EMM** | **Adjusted Tukey p-value** |
| --- | --- | --- | --- | --- |
| superpathway of polyamine biosynthesis II | *Echium vulgare* | 0.190311 | 0.219783 | 0.408945 |
|  | *Lotus corniculatus* | 0.185178 | 0.220614 | 0.217001 |
|  | *Festuca rubra* | 0.195387 | 0.140837 | 0.01203 |
| L-tryptophan degradation IX | *Echium vulgare* | 0.046249 | 0.071898 | 0.097672 |
|  | *Lotus corniculatus* | 0.047226 | 0.076001 | 0.045646 |
|  | *Festuca rubra* | 0.047005 | 0.045205 | 0.999967 |

| L-cysteine biosynthesis VI (from L-methionine) | *Echium vulgare* | 0.216868 | 0.267483 | 0.129383 |
| --- | --- | --- | --- | --- |
|  | *Lotus corniculatus* | 0.232052 | 0.264743 | 0.567715 |
|  | *Festuca rubra* | 0.227621 | 0.299282 | 0.009424 |
| assimilatory sulfate reduction I | *Echium vulgare* | 0.505711 | 0.526847 | 0.91345 |
|  | *Lotus corniculatus* | 0.507957 | 0.542193 | 0.584501 |
|  | *Festuca rubra* | 0.506137 | 0.544697 | 0.4553 |
| superpathway of allantoin degradation in plants | *Echium vulgare* | 0.069445 | 0.022707 | 0.000142 |
|  | *Lotus corniculatus* | 0.070567 | 0.021723 | 0.000142 |
|  | *Festuca rubra* | 0.062731 | 0.022744 | 0.000173 |
| photorespiration | *Echium vulgare* | 0.257303 | 0.251956 | 0.999944 |
|  | *Lotus corniculatus* | 0.258857 | 0.24248 | 0.986869 |
|  | *Festuca rubra* | 0.248421 | 0.209849 | 0.654858 |
| superpathway of geranylgeranyl diphosphate biosynthesis II (via MEP) | *Echium vulgare* | 0.409733 | 0.392068 | 0.169556 |
|  | *Lotus corniculatus* | 0.412038 | 0.378748 | 0.000684 |
|  | *Festuca rubra* | 0.40472 | 0.39749 | 0.917206 |
| fatty acid &beta;-oxidation II (plant peroxisome) | *Echium vulgare* | 0.783529 | 0.684238 | 0.125960 |
|  | *Lotus corniculatus* | 0.788524 | 0.687587 | 0.115112 |
|  | *Festuca rubra* | 0.764345 | 0.686055 | 0.342593 |
| phytate degradation I | *Echium vulgare* | 0.001521 | 0.001398 | 0.999506 |
|  | *Lotus corniculatus* | 0.001907 | 0.001043 | 0.218164 |
|  | *Festuca rubra* | 0.002109 | 0.000818 | 0.016126 |
| superpathway of putrescine biosynthesis | *Echium vulgare* | 0.254291 | 0.311271 | 0.038122 |
|  | *Lotus corniculatus* | 0.254353 | 0.302316 | 0.118130 |
|  | *Festuca rubra* | 0.256451 | 0.273195 | 0.941815 |

Legend: NS, non-sterilized substrate; S, sterilized substrate; EMM, estimated marginal mean

**TABLE S7** Substrate parameters and their relevance to the microbiome

| **Substrate parameter** | **Relevance to the microbiome** | **Reference** |
| --- | --- | --- |
| pH | Determines the availability of nutrients and the enzymatic activity of microorganisms; differences in bacterial communities are strongly correlated with soil pH at various scales | Fierer & Jackson 2006; Mitsuta et al. 2025 |
| NO3^−^-N | The main growth-limiting factor; microorganisms can indirectly influence nitrogen uptake by plants by affecting root characteristics or inducing physiological responses | Koch & Sessitsch 2024 |
| P | A key element in nutrient cycling; microorganisms can indirectly influence phosphorus uptake by plants through mineralization, dissolution, and transformation of inorganic and organic phosphorus that is difficult to access in soils | Feng & Chen 2024 |
| K | Potassium homeostasis is essential for bacterial survival, affecting, e.g., enzyme activation, signaling, and osmoregulation | Stautz et al. 2021 |
| Ca | Can increase bacterial network complexity and shift composition; a key factor in the growth and activity of fungi and bacteria, e.g., in surface adhering, biofilm-forming bacterial and fungal lignin-degrading enzymes | Shabtai et al. 2023 |
| Mg | Important nutrient for bacterial growth and cell maintenance; affects biofilm development with respect to, e.g., cell attachment; as an external add-on, e.g., as a fertilizer, can increase soil microbial biomass, enzyme activity, and bacterial community composition | Wang et al. 2019; Yang et al. 2021 |
| Cl | Changes in Cl^-^ availability can shape microbial community structure through the influence on soil enzyme activities; fungal communities are more affected by Cl level shifts | Gryndler et al. 2008 |
| Na & NaCl salinity | Selecting factors for haloalkalitolerant and haloalkaliphilic bacterial communities; combined with pH, are the edaphic factors influencing both bacterial diversity and activity | Mucsi et al. 2024 |
| organic matter | Strong positive driver of microbial biomass and cross-domain microbial interactions | Heisey et al. 2022 |
| bulk density | Increased bulk density generally reduces microbial richness, biomass, and alters community composition, although these reports are not conclusive | Longepierre et al. 2021; Yu et al. 2021 |

**Figure S1** Rarefaction curves based on the Shannon diversity index for bacterial communities (16S rRNA gene sequencing). Each curve represents an individual sample and shows the relationship between sequencing depth and estimated within-sample (alpha) diversity

**Figure S2** Rarefaction curves based on the Shannon diversity index for fungal communities (ITS amplicon sequencing). Each curve represents an individual sample and shows the relationship between sequencing depth and estimated within-sample (alpha) diversity

**Figure S3** Alpha diversity (Shannon index) of bacterial communities across experimental groups. Statistical comparisons were performed using the Kruskal–Wallis test followed by pairwise comparisons with Benjamini–Hochberg correction

**Figure S4** Alpha diversity of fungal communities across experimental groups. Statistical comparisons were performed using the Kruskal–Wallis test followed by pairwise comparisons with Benjamini–Hochberg correction

**FIGURE S5** LEfSe identification of discriminant bacterial genera associated with experimental groups

**FIGURE S6** LEfSe identification of discriminant fungal genera associated with experimental groups

**Supplementary Dataset.xlsx** This file contains two datasets: **Dataset 1**, comprising SparCC correlation results for microbial co-occurrence network analysis across six experimental groups, and **Dataset 2**, comprising PICRUSt2-predicted metabolic pathway abundances across individual biological replicates. The datasets are provided in separate worksheets.
